# Neurogenesis redirects β-catenin from adherens junctions to the nucleus to promote axonal growth

**DOI:** 10.1242/dev.201651

**Published:** 2023-08-24

**Authors:** Antonio Herrera, Anghara Menendez, Andrea Ochoa, Lídia Bardia, Julien Colombelli, Sebastian Pons

**Affiliations:** ^1^ Instituto de Biología Molecular de Barcelona (IBMB-CSIC), Parc Científic de Barcelona, Baldiri Reixac 10-12, Barcelona 08028, Spain; ^2^ Institute for Research in Biomedicine (IRB Barcelona), Barcelona Institute of Science and Technology (BIST), Baldiri Reixac 10, Barcelona 08028, Spain

**Keywords:** Spinal cord, Neuroepithelium, Commissural neurons, Neural differentiation, Cell polarity, Adherens junctions, N-cadherin, β-catenin, Tcf/Lef transcription, Chick

## Abstract

Here, we show that, in the developing spinal cord, after the early Wnt-mediated Tcf transcription activation that confers dorsal identity to neural stem cells, neurogenesis redirects β-catenin from the adherens junctions to the nucleus to stimulate Tcf-dependent transcription in a Wnt-independent manner. This new β-catenin activity regulates genes implicated in several aspects of contralateral axon growth, including axon guidance and adhesion. Using live imaging of *ex-vivo* chick neural tube, we showed that the nuclear accumulation of β-catenin and the rise in Tcf-dependent transcription both initiate before the dismantling of the adherens junctions and remain during the axon elongation process. Notably, we demonstrated that β-catenin activity in post-mitotic cells depends on *TCF7L2* and is central to spinal commissural axon growth. Together, our results reveal Wnt-independent Tcf/β-catenin regulation of genes that control the growth and guidance of commissural axons in chick spinal cord.

## Teaser

**Summary:** Neurogenesis redirects β-catenin from the adherens junctions to the nucleus to stimulate genes that control the growth and guidance of commissural axons in developing chick spinal cord.

## INTRODUCTION

During spinal cord (SC) development, canonical Wnt signaling has an essential role in dorsoventral patterning and proliferation of neural stem cells (NSCs) (Muroyama et al., 2002; Alvarez-Medina et al., 2008; Andrews et al., 2019). Roof plate-secreted Wnts bind to Frizzled receptors and co-receptor LRP5/6 at the membrane of NSCs to release β-catenin from the Axin/GSK3β/APC destruction complex, from which it is translocated into the nucleus. Once in the nucleus, β-catenin forms complexes with Tcf/Lef transcription factors, which bind directly to genomic Tcf/Lef response elements to induce the expression of their target genes (Clevers, 2006; Nusse and Clevers, 2017). However, β-catenin also plays an important role in apical–basal cell polarity of NSCs by contributing to the maturation of N-cadherin and, consequently, to the establishment of apical adherens junctions (AJs) (Baum and Georgiou, 2011; Herrera et al., 2021).

Regionalization of the neural tube (NT) begins early in neurodevelopment, while NSCs proliferate symmetrically in a self-expanding mode. Later on, and in association with the onset of neurogenesis, NSCs change their mode of division to generate the first committed neurons (Gotz and Huttner, 2005; Saade et al., 2013). After neurogenic divisions, post-mitotic NSCs diminish N-cadherin expression and increase *TUBB3* expression before detaching from the proliferative ventricle in a process known as apical abscission (Rousso et al., 2012; Das and Storey, 2014; Sagner et al., 2018). During the first wave of neurogenesis, 11 different populations of spinal neurons are generated: six dorsal interneuron populations (dI1-dI6), three ventral interneuron populations (V0-V3) and a large number of motor neurons (Helms and Johnson, 2003; Lai et al., 2016). The interneuron populations can reach ipsilateral or contralateral targets; these last neurons are known as commissural neurons. Their axons grow towards the floor plate (FP), cross the ventral midline (commissure) and turn following the FP. Then, a large proportion of them deviate to follow the ventral funiculus (VF) or lateral funiculus (LF) until reaching their targets (Kadison and Kaprielian, 2004; Chédotal, 2019). Axon guidance signals, including Netrin/DCC, Slit/Robo, sonic hedgehog (Shh)/BOC, EPHB3/EPHA4 and Wnts/Fzd3, work together to promote the growing and guiding of commissural axons. FP Shh and netrin 1 guide commissural axons to cross the midline by binding to BOC and DCC on pre-crossing commissural axons (Fazeli et al., 1997; da Silva et al., 2018). After crossing the midline, FP-secreted Slit proteins repel commissural growth cones by activating Robo1 and Robo2 receptors, preventing them from re-entering the midline (Long et al., 2004; Reeber et al., 2008; Jaworski et al., 2010). Various Wnt proteins induce the anterior turning and growth of post-crossing commissural axons through Fzd3 receptor (Lyuksyutova et al., 2003; Onishi and Zou, 2017). This is part of the planar cell polarity (PCP) signaling, a pathway that is independent of β-catenin/Tcf/Lef proteins. In addition, guidance pathways coordinate with adhesion molecules such as N-cadherin and integrins to promote directional remodeling of the actin cytoskeleton during axonal growth and pathfinding (Rhee et al., 2002; Myers and Gomez, 2011).

Here, we combine the expression of Cre recombinase under the control of a 229 bp proximal element of the *TUBB3* gene promoter region with multiple LoxP vectors to dissect post-mitotic from proliferative β-catenin/Tcf/Lef-mediated transcription. We demonstrate that, after the initial activation of Tcf-dependent transcription by Wnt proteins that confer dorsal identity to NSCs of chick SC, Tcf-dependent transcription is activated again by a Wnt-independent mechanism in differentiating SC commissural neurons. This second transcription activation controls the expression of genes involved in axon guidance and adhesion required for contralateral axon growth. We used live imaging of *ex vivo* chick NT to show that β-catenin accumulates in the nucleus just before the dismantling of the AJs that precedes the apical abscission. Nuclear β-catenin then activates Tcf/Lef-dependent transcription, which remains high during axon elongation. We also show that the downregulation of *TCF7L2* prevents post-mitotic Tcf transcription and severely impairs spinal commissural axon growth. Together, our results reveal a Wnt-independent Tcf/β-catenin regulation of genes that control the growth and guidance of commissural axons in the chick SC.

## RESULTS

### Tcf/Lef-dependent transcription is activated during commissural differentiation in the chick SC

**Fig. 1.**
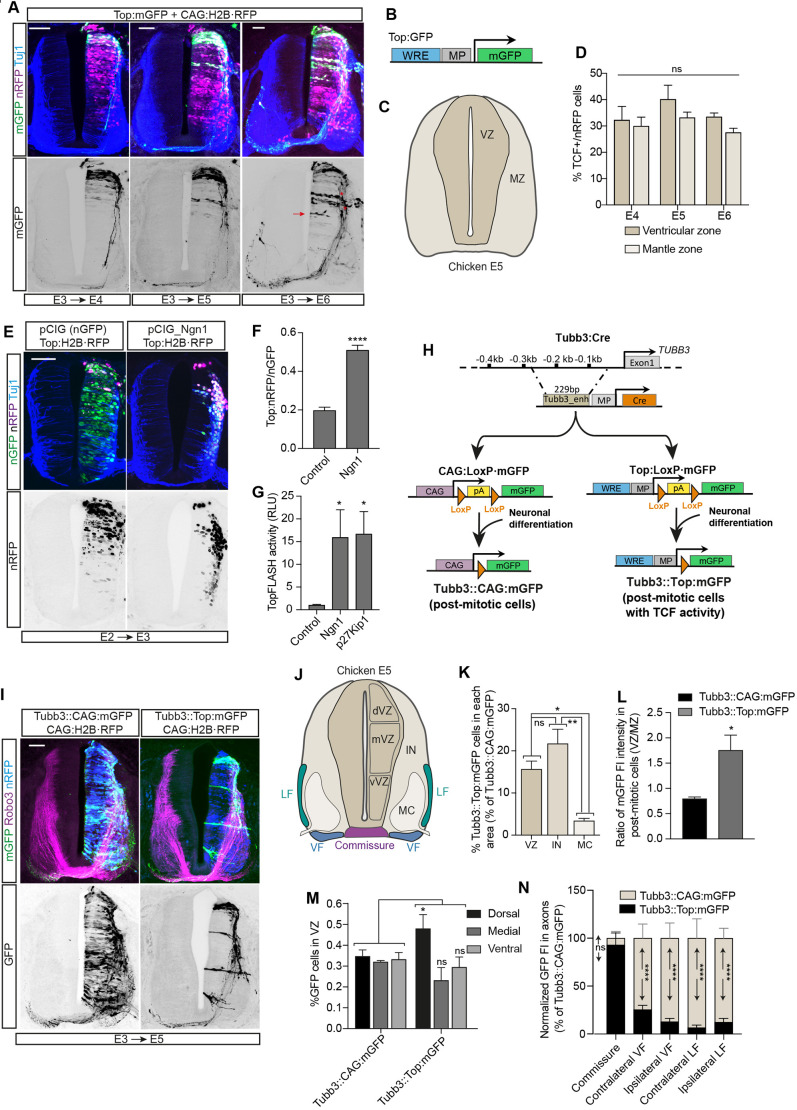
**Tcf/Lef-dependent transcription is activated during commissural differentiation in the chick spinal cord (SC).** (A) Embryonic day (E)3 chick neural tubes (NTs) electroporated for 24, 48 or 72 h [E3+24 or 48 or 72 h post-electroporation (hpe)] with Top:mGFP and CAG:H2B·RFP, which show Tcf activity (membrane; green) and transfection (nuclear; magenta), respectively. Transverse slices were stained with anti-Tubb3 (Tuj1) antibody (labels neurons; blue). The red arrow points to ventricular Top^+^ cells out of the Wnt response domain. Asterisks mark dorsal mantle zone (MZ) Top^+^ neurons. Scale bars: 50 μm. (B) Vector used to detect Tcf/Lef activity. MP, minimal promoter; WRE, Wnt response elements. (C) Scheme of areas occupied by the MZ and the ventricular zone (VZ) in an E5 transverse slice of a thoracic chick SC. (D) The percentage of Top^+^ cells among the electroporated ones in the MZ and VZ at 24, 48 or 72 hpe. Each data point=6 slices. (E) E2 chick NTs electroporated for 24 h (E2+24 hpe) with Top:H2B·RFP (nuclear; magenta) plus Ngn1 or its control vector pCIG (nuclear; green). E3 slices were stained with anti-Tubb3 antibody (blue). Scale bar: 50 μm. (F) The percentage of Top^+^ cells among the electroporated ones in control and Ngn1 transfected embryos. Each data point=5 slices. (G) TopFlash luciferase activity from E2 embryos electroporated for 24 h (E2+24 hpe) with Top-Flash plus control, Ngn1 or p27^Kip1^. Each data point=5 slices. RLU, relative light units. (H) Scheme of the CRE/Lox-based vector system created to monitor or manipulate Tcf activity in post-mitotic neurons. The vectors express mGFP cloned in the second slot of a bicistronic arrangement. pA, poly-adenylating sequence. (I) E3 chick NTs electroporated for 48 h (E3+24 hpe) with Tubb3::CAG:mGFP (shows all transfected neurons, membrane; green) or Tubb3::Top:mGFP (shows Tcf activity in transfected neurons, membrane; green) and CAG:H2B·RFP (shows nucleus; blue). E5 slices were stained with anti-Robo3 antibody (stains commissural axons from the cell body to commissure, magenta). Scale bar: 50 μm. (J) Scheme of the VZ (subdivided in dorsal, medial and ventral areas), interneurons (IN), motor column (MC), lateral funiculus (LF), ventral funiculus (VF) and the commissure in E5 chick SC. dVZ, dorsal ventricular zone; mVZ, medial ventricular zone. (K) The percentage of Top^+^ neurons (Tubb3::Top:mGFP) from all transfected neurons (Tubb3::CAG:mGFP) in the VZ, IN and MC (E3+24 hpe). Each data point=3 slices. (L) Ratio between the mGFP fluorescence emitted by neurons in the VZ and MZ, either under the Top promoter or under a constitutive promoter (CAG; E3+24 h). Each data point=10 slices for control and 14 for Top. (M) The percentage of Top^+^ neurons (Tubb::Top:mGFP) from all transfected neurons (Tubb3::CAG:mGFP) in the dorsal, medial and ventral regions of the VZ of E3 embryos transfected for 24 h (E3+24 hpe). Each data point=4 slices for control and 11 for Top. (N) Relative fluorescence intensity (FI) of mGFP measured in the axons of commissure, contralateral VF, ipsilateral VF, contralateral LF and ipsilateral LF from E3 embryos electroporated for 48 h (E3+24 hpe), comparing Tubb3::Top:mGFP with Tubb::CAG:mGFP. Each data point=11 slices for control and 14 for Top. Bar graphs show the mean±s.e.m. *P<0.05, **P<0.01, ****P<0.0001; ns, non-significant [unpaired two-tailed t-test (F,L), one-way ANOVA plus Dunn's multi-comparisons test (G), one-way ANOVA plus Tukey's multi-comparisons test (K), two-way ANOVA plus Sidak's multi-comparisons test (D,M), two-way ANOVA plus Tukey's multi-comparisons test (N)].

*In ovo* electroporation of chick NTs permits genetic manipulations that can be followed during neural development. We used the thoracic chick SC to study the hitherto unknown Tcf/Lef-dependent transcription activation that occurs coincident with the initiation of neural differentiation. Using a construct expressing membrane GFP (mGFP) under the Top promoter, containing five copies of a Tcf/Lef response element (WRE) and a thymidine kinase (TK) minimal promoter, we electroporated the NTs of embryonic day (E)3 (3 days post-conception) chick embryos and followed them for 24, 48 or 72 h. Notably, we observed that, in E4 chick SCs, in addition to the dorsal group of NSCs conforming to the Wnt response domain, Tcf/Lef-mediated transcription was also active (Top^+^) in dorsal cells with neuronal morphology. As neurogenesis extended to more ventral regions on E5 and E6, the presence of Top^+^ cells with signs of neural differentiation also extended ventrally (Fig. 1A,B). Interestingly, many of these Top^+^ neurons extended axons to the contralateral side through the commissure. During their differentiation, neurons migrated laterally to form the mantle zone (MZ) basal to the ventricular zone (VZ; Fig. 1C). Interestingly, we observed that the percentage of Top^+^ cells remained constant from E4 to E6. Moreover, no differences were observed between the VZ and the MZ, suggesting that Tcf/Lef-mediated transcription remained active throughout the neural differentiation process (Fig. 1D). The dorsal Wnt response domain is already significantly reduced at E5 (Agalliu et al., 2009) (Fig. S1A). However, to ensure that the observed Tcf/Lef activity in neurons was not inherited from Wnt-responsive NSCs, we used a Top vector that drives the expression of a GFP containing a PEST sequence at its C-terminus, which reduced its half-life from 20 h to 2 h (Li et al., 1998; He et al., 2019). Again, we observed Tcf/Lef activity in ventricular progenitor cells and differentiated neurons (Fig. S1B). Neurogenin 1 (*NGN1*) is a pro-neural gene that induces premature neuronal differentiation and commissural dI2 subtype specification in SC (Gowan et al., 2001; Alaynick et al., 2011; Le Dreau et al., 2018). We observed that transfection of Ngn1 for 24 h in E2 chick NTs increased the percentage of Top^+^ cells in a cell-autonomous manner (Fig. 1E,F). Moreover, transfection of either Ngn1 or p27^Kip1^, a cell cycle repressor that induces premature neural differentiation, significantly increased the activity of TopFlash, a Tcf/Lef luciferase reporter (Fig. 1G). Henceforth, the upregulation of Tcf/Lef-dependent transcription observed during neural differentiation will be referred to as ‘differentiation-associated Tcf/Lef transcription’ (DATT). Using an enhancer element from the *TUBB3* gene promoter (Tubb3_enh) (Bergsland et al., 2011), we generated Cre-Lox-based vectors to study and manipulate DATT without altering the pathway in NSCs. In addition, we created two groups of vectors in which the transcription of mGFP, driven either by the CAG promoter (b-actin promoter with the CMV^E1A^ enhancer) or the Top promoter, was blocked by a floxed poly-adenylating sequence (pA; Fig. 1H). The vectors showed good expression and low leakage (Fig. S1C). Co-transfection of Tubb3:Cre with CAG:LoxP·mGFP (Tubb3::CAG:mGFP) showed all the differentiating neurons among the transfected cells (transfection was monitored with H2B·RFP), whereas co-transfection of Tubb3:Cre with Top:LoxP·mGFP (Tubb3::Top:mGFP) revealed only the Top^+^ differentiating neurons (Fig. 1I, Fig. S1D). In E5 chick SCs, the MZ can be further divided into two areas containing the interneurons (IN) and the motor column (MC) (Fig. 1J). We used anti-Robo3, which labels commissural axons from the cell body to commissure (Fig. 1I), to delimitate the IN, the MC, the VZ and the three main axonal tracts containing commissural interneuron projections: the commissure, the contralateral ventral funiculus (VF) and the contralateral lateral funiculus (LF; Fig. 1J) (Wu et al., 2019). In E5 chick SCs that had been transfected for 24 h, the percentage of Top^+^ among post-mitotic cells was significantly higher in the VZ and the IN than in the MC (15.7±3.2% in VZ, 21.77±5.82 in IN and 3.45±0.89% in MC; Fig. 1I,K). In addition, the VZ/MZ GFP ratio was significantly higher in Tubb3::Top:mGFP than in Tubb3::CAG:mGFP embryos, indicating that although the CAG activity remained constant during differentiation, the Top activity was higher while the future neurons were still in the VZ (Fig. 1I,L). Compared with total transfected neurons (Tubb3::CAG:mGFP), Top^+^ neurons (Tubb3::Top:mGFP) were more abundant in the dorsal region than in the medial and ventral regions of the VZ (Fig. 1M). This result demonstrated that DATT was more abundant in the dorsal SC, but present at other dorsoventral levels, suggesting that DATT was mostly present in commissural neurons. To test that, we compared GFP fluorescence intensity in the different axonal tracks of Tubb3::CAG:mGFP and Tubb3::Top:mGFP embryos. Notably, almost 100% of transfected axons were Top^+^ in the commissure, whereas this percentage was very low in the other tracts, except for the ventral contralateral track, in which it was moderate (Fig. 1N). These results confirmed that the observed Top^+^ neurons were mostly commissural neurons in which Tcf/Lef-dependent transcription was activated during differentiation.

### DATT is required for commissural outgrowth and pathfinding in developing SC

**Fig. 2.**
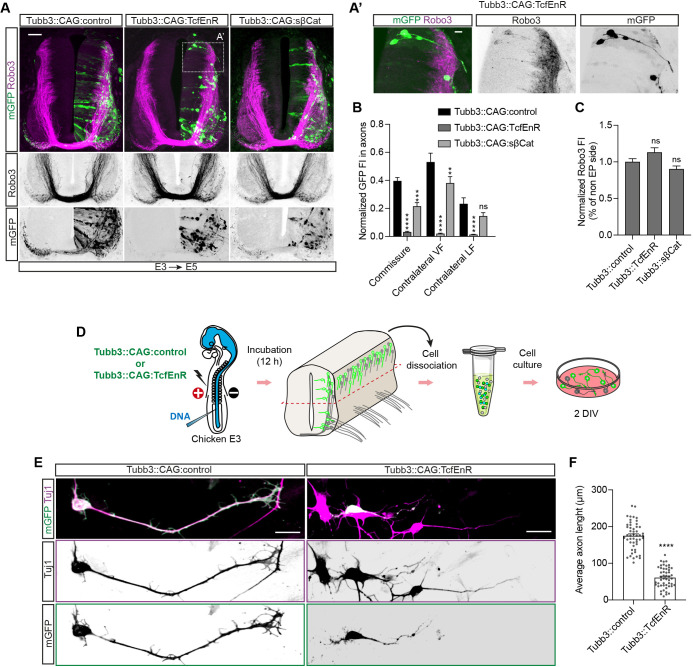
**Tcf/Lef-dependent transcription is required for commissural outgrowth and pathfinding in developing SC.** (A) E3 chick NTs electroporated for 48 h (E3+24 hpe) with Tubb3::CAG:control, Tubb3::CAG:TcfEnR or Tubb3::CAG:sβ-Cat. mGFP indicates transfected neurons (membrane; green). Transverse sections were stained with an anti-Robo3 antibody (magenta). (A′) Enlargement of the area enclosed by the dotted line in A. Scale bars: 50 μm (A) and 10 μm (A′). (B) Normalized FI of mGFP measured in the axons of commissure, contralateral VF or contralateral LF from E3 embryos electroporated for 48 h (E3+24 h) with Tubb3::CAG:control, Tubb3::CAG:TcfEnR or Tubb3::CAG:sβ-Cat. Each data point=14 slices for control, 21 for TcfEnR and 20 for sβ-Cat (C) Ratio between electroporated and non-electroporated sides of normalized Tubb3 FI from E3 embryos transfected for 24 h (E3+24 hpe) with Tubb3::CAG:control, Tubb3::CAG:TcfEnR or Tubb3::CAG:sβ-Cat. Each data point=3 slices for control, 7 for TcfEnR or 5 for sβ-Cat. EP, electroporated. (D) Scheme of the procedure followed to prepare cultures of interneurons transfected with Tubb3::CAG:control or Tubb3::CAG:TcfEnR. DIV, days *in vitro*. (E) Representative images of interneurons transfected with Tubb3::CAG:control or Tubb3::CAG:TcfEnR stained with anti-Tubb3 (Tuj1) antibody (magenta), mGFP indicates transfection (membrane; green). Scale bars: 15 μm. (F) Average axon length of cultured interneurons transfected with Tubb3::CAG:control or Tubb3::CAG:TcfEnR after 2 DIV. Each point=50 axons from three culture wells. Bar graphs show the mean±s.e.m. **P<0.01, ***P<0.001, ****P<0.0001; ns, non-significant [unpaired two-tailed *t*-test (F), one-way ANOVA plus Dunnett's multiple comparisons test (C), two-way ANOVA plus Dunnett's multiple comparisons test (B)].

In the absence of nuclear β-catenin, Tcf/Lefs act as transcriptional repressors by binding to Groucho/TLE proteins (Daniels and Weis, 2005). Expression of β-catenin^S33Y^, a stable form of β-catenin (sβ-Cat), induces potent activation of Tcf-dependent transcription in chick NT, whereas TcfEnR, a fusion of Tcf3 HMG-box (Tcf3 DNA-binding domain) with the transcriptional repressor engrailed (Eng), drastically reduces it (Herrera et al., 2014). We used the bicistronic CAG:LoxP·mGFP vector to express either TcfEnR or sβ-Cat, which combined with Tubb3:Cre produced Tubb3::CAG:TcfEnR and Tubb3::CAG:sβ-Cat, respectively (Fig. S2A). We electroporated E3 chick NTs for 24 h with Tubb3::CAG:TcfEnR or Tubb3::CAG:sβ-Cat. mGFP showed the cell body and projections of transfected cells, whereas anti-Robo3 stained commissural axons. Suppression or sustained activation of Tcf/Lef-dependent transcription reduced commissural axons. However, the effect of sβ-Cat was very moderate compared with the severe reduction caused by TcfEnR (Fig. 2A,B). TcfEnR expression caused ectopic exit of commissural axons through the dorsal root (Fig. 2A′). These effects could be cell autonomous, as total Robo3 expression was unaffected in the descending commissural tracts (Fig. 2C). To evaluate whether Tcf-dependent transcription was required for axon outgrowth, we transfected E3 chick embryos with TcfEnR (Tubb3::CAG:TcfEnR) or control (Tubb3::CAG:mGFP) and, 12 h later, dissected the dorsal NTs to prepare interneuron cultures (Langlois et al., 2010) (Fig. 2D). Notably, we observed that suppression of Tcf/Lef transcription significantly reduced the length of axons after 2 days in culture (Fig. 2E,F).

### DATT promotes commissural axon elongation and pathfinding through adhesion and axon guidance pathways

**Fig. 3.**
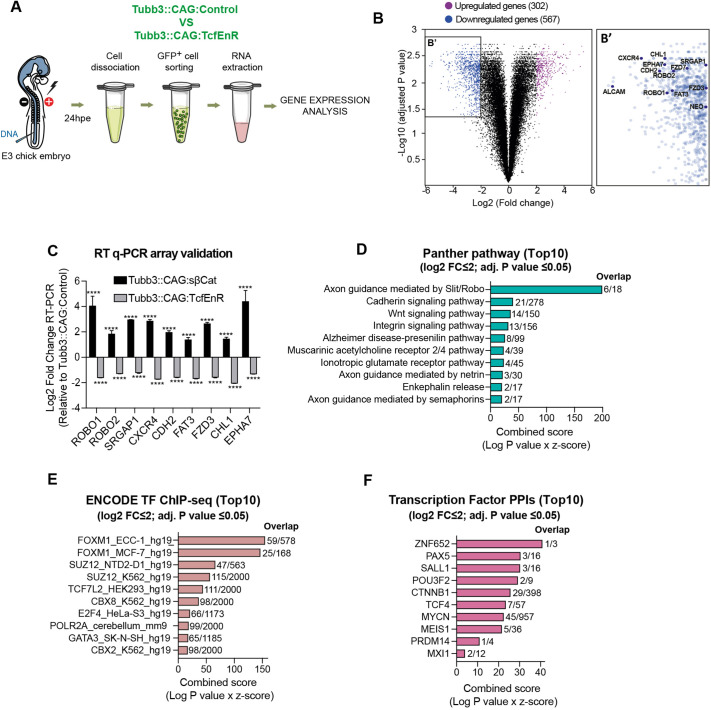
**Differentiation-associated Tcf/Lef transcription (DATT) promotes commissural axon elongation and pathfinding through adhesion and axon guidance pathways.** (A) Scheme of the procedure followed to study the Tcf/Lef-dependent genes that are active during neural differentiation. (B) Volcano plot showing the corrected gene expression (total abundance and relative variation), comparing embryos transfected with Tubb3::CAG:control with those transfected with Tubb3::CAG:TcfEnR. Variations superior to fourfold with *q*-values <0.05 were considered significant. Downregulated and upregulated transcripts are highlighted in blue and magenta, respectively. [log2 fold change (FC)] ≥2, with adjusted *P*-value ≤0.05. (B′) Magnification of the downregulated genes, highlighting a group of 11 manually selected genes involved in diverse aspects of neural differentiation. Each data point=3 independent PCRs. (C) Validation of the experimental approach by assessing the expression of a group of very-relevant genes by RT-qPCR, comparing embryos transfected with Tubb::CAG:TcfEnR (Tcf/Lef activity suppressed in neurons) with those transfected with Tubb3::CAG:sβ-Cat (Tcf/Lef activity constitutively activated in neurons). (D) Combined score of the top ten Panther pathways that were changed by Tcf/Lef activity suppression in neurons. (E) Combined score of the top ten transcription factors intervening based on the ENCODE TF chromatin immunoprecipitation with DNA sequencing (ChIP-seq) database. (F) Combined score of the top ten proteins interacting with the implicated transcription factors based on the Transcription Factor PPIs database. Bar graphs show the mean±s.e.m. ****P<0.0001 (multiple *t*-tests and two-way ANOVA plus Dunnett's multiple comparisons test).

**Fig. 4.**
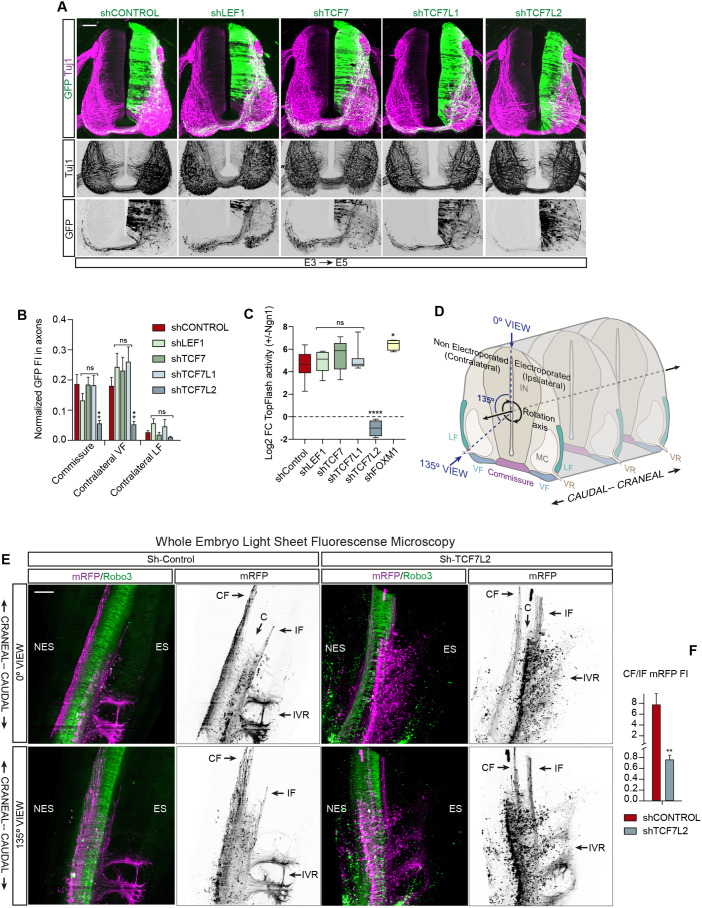
**DATT is mediated by TCF7L2.** (A) E3 chick NTs electroporated for 48 h (E3+24 hpe) with GFP-expressing pSHIN vectors encoding shRNAs targeting LEF1, TCF7, TCF7L1 or TCF7L2. SC transverse sections were stained with anti-Tubb3 (Tuj1) antibody (magenta), and GFP indicates transfection (green). Scale bar: 50 μm. (B) Normalized FI of mGFP measured in the axons of commissure, contralateral VF or contralateral LF from E3 chick NTs electroporated for 48 h (E3+24 hpe) with pSHIN vectors encoding shRNAs against LEF1, TCF7, TCF7L1 or TCF7L2. Each data point=14 slices for control, 11 for LEF1, 13 for TCF7, 9 for TCFL1 and 12 for TCFL2 (C) Ratio variation (log2 FC) of TopFlash activity (luciferase) in embryos transfected or not with Ngn1 and pSHIN vectors encoding shRNAs against LEF1, TCF7, TCF7L1, TCF7L2 or FOXM1. Each data point=16 embryos for control, 6 for LEF1, 7 for TCF7, 7 for TCFL1, 4 for TCFL2 and 4 for FOXM1. (D) Scheme of an E5 SC to assist with panel E and 3D image interpretation. IN, interneurons; LF, lateral funiculus; MC, motor column; VF, ventral funiculus; VR, ventral root. (E) Maximum-intensity projections using 0° and 135° perspectives of 360° images generated from cleared E3 chick embryos electroporated for 24 h (E3+24 hpe) with pCS2-mRFP plus pSHIN-shTCF7L2 or pSHIN-shControl, which were whole-mount immunolabeled with anti-RFP (magenta) and anti-Robo3 (green) antibodies. Volume imaging was performed through light-sheet fluorescence microscopy at 9.6×. Scale bar: 250 μm. C, commissure; CF, contralateral funiculus; ES, electroporated side; IF, ipsilateral funiculus; IVR, ipsilateral ventral rods; NES, non-electroporated side. (F) Ratio of FI of mRFP measured in the axons of ipsilateral and contralateral VF measured in two embryos for each condition. Bar graphs show the mean±s.e.m. *P<0.05, **P<0.01, ****P<0.0001; ns, non-significant [unpaired two-tailed *t*-test (F), one-way ANOVA plus Dunnett's multiple comparisons test (C), two-way ANOVA plus Dunnett's multiple comparisons test (B)].

To study the Tcf-dependent genes that were active during commissural neuron differentiation, we transfected E3 chick embryos for 24 h to produce either Tubb3::CAG:TcfEnR (suppression of Tcf-dependent transcription in neurons) or Tubb3::CAG:control (controls) and cell sorted the GFP^+^ cells. Then, we used Affymetrix GeneChip arrays to study the transcriptomes (Fig. 3A). Comparing Tubb3::CAG:TcfEnR transfected embryos with controls, 302 genes were upregulated, and 567 were downregulated (Table S1). The Affymettrix data are in Gene Expression Omnibus (GEO), accession number GSE234518 (see Table S2 for metadata). We were mostly interested in the group of downregulated genes, assuming that these genes were the ones that, either directly or indirectly, were activated by DATT (Fig. 3B). To validate the rationale of this approach, we manually selected a group of genes involved in different aspects of axon outgrowth and guidance (Fig. 3B′), and studied their expression by real-time quantitative PCR (RT-qPCR), comparing Tubb3::CAG:TcfEnR with Tubb3::CAG:sβ-Cat, where the pathway was activated by sβ-Cat. Consistently, the group of genes that were downregulated in Tubb3::CAG:TcfEnR embryos were enhanced by sβ-Cat, confirming that all the genes in this group were β-catenin/Tcf-dependent targets (Fig. 3C). We further computationally analyzed the group of downregulated genes [log2 fold change (FC) ≤2 with adjusted *P*-value ≤0.05] by Gene Ontology (GO) enrichment analysis plus PANTHER classification system to find the pathways affected (Fig. 3D) (Ashburner et al., 2000; Mi et al., 2019; Gene-Ontology-Consortium, 2021). Additionally, we used Enrichr to determine the transcription factors intervening (ENCODE TF database; Fig. 3E) and the proteins associated with those transcription factors (Transcription Factor PPIs database; Fig. 3F) (Chenet al., 2013; Kuleshov et al., 2016; Xie et al., 2021). PANTHER predicted the signaling pathways of cadherins, integrins and Wnt proteins to be regulated by DATT with high scores; however, the Slit/Robo axon guidance pathway received the highest score. Although receiving lower scores, the expression of other genes involved in the axon guidance pathways mediated by netrin and semaphorins was also significantly downregulated in Tubb3::CAG:TcfEnR neurons. The ENCODE TF database predicted that TCF7L2 (Tcf4) was one of the main transcription factors mediating Tcf-dependent transcription in this process. So, we used pSHIN, a GFP-expressing vector, to clone short hairpin inhibitory RNAs (shRNAs) against the different Tcf/Lef transcription factors expressed in E4 chick NTs – LEF1, TCF7, TCF7L1 and TCF7L2 (Fig. S2B,C) – transfected them into E3 chick NTs for 48 h, and stained sections with anti-Tuj1 (Tubb3) (Fig. 4A) or anti-Robo3 (Fig. S2D) to study the presence of GFP^+^ axons in contralateral axonal tracks. Notably, the presence of GFP^+^ fibers in the commissure and the contralateral VF was significantly reduced after *TCF7L2* suppression compared with that in the controls or the other knockdowns (Fig. 4B). This effect was not due to the suppression of dorsal patterning previously reported by Wnt/β-catenin pathway inhibition (Fig. S2E). We transfected E3 embryos with the mentioned shRNAs, plus TopFlash reporter and Ngn1 or control vector. As expected, expression of Ngn1 increased Tcf-dependent transcription; however, this increase disappeared with *TCF7L2* suppression (Fig. 4C). Among Tcf/Lef transcription factors, TCF7L2 received the highest score by the ENCODE TF database in mediating DATT; however, the absolute highest score was given to FOXM1, a transcription factor reported to be required for neuronal differentiation (Ueno et al., 2008). Nevertheless, suppression of *FOXM1* did not impede the effect of Ngn1 on Tcf-dependent transcription (Fig. 4C). Altogether, these results confirmed that DATT regulates the transcription of a set of genes involved in commissural axon elongation and guidance specifically through the TCF7L2 transcription factor. To further assess the effect of *TCF7L2* suppression, we transfected E3 chick embryos for 24 h [E3+24 h post electroporation (hpe)] with pCS2-mRFP plus pSHIN-shTCF7L2 or pSHIN-shControl, whole-mount immunolabeled them with anti-RFP and anti-Robo3 antibodies, cleared them optically and performed volume imaging through light-sheet fluorescence microscopy. Bearing in mind that we only electroporate one side of the neural tube, which we consider to be ipsilateral (Fig. 4D), this technique is suitable to reveal perturbations in the ratio of axons occupying ipsilateral versus contralateral axonal tracts. In accordance with the 2D images shown in Fig. 4A, the 3D images generated from light-sheet microscopy confirmed that *TCF7L2* knockdown significantly decreased the ratio of axons that crossed and followed the contralateral tract with respect to the ones that followed the ipsilateral tract (7.75±0.35 in control versus 0.79±0.02 in TCF7L2 knockdown; Fig. 4E,F, Movies 5 and 6). Concomitant with the decrease in contralateral axons, an important accumulation of GFP was observed in the somas of pSHIN-shTCF7L2 transfected cells, but not in the ipsilateral tracts, indicating a defect in the growth of commissural axons rather than redirection towards the ipsilateral tracks.

### DATT is Frizzled independent and begins before apical abscission

**Fig. 5.**
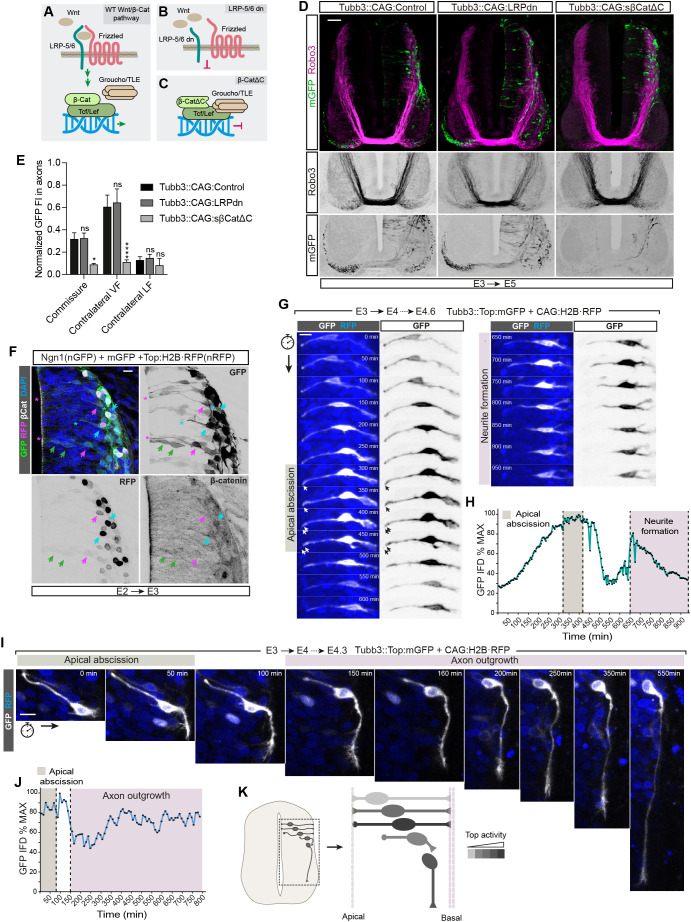
**DATT is Frizzled independent and begins before apical abscission.** (A-C) Schemes of the wild-type canonical Wnt pathway (A), dominant-negative LRP5/6 with a C-terminal deletion (B) and β-catenin with a C-terminal deletion (C). (D) E3 chick NTs electroporated for 48 h (E3+24 hpe) with Tubb3::CAG:control, Tubb3::CAG:LRPdn or Tubb3::CAG:sβ-CatΔC. Transverse sections were stained with anti-Robo3 antibody (magenta). mGFP is expressed by transfected neurons (membrane; green). Scale bar: 50 μm. (E) Normalized mGFP FI measured in the axons of the commissure, contralateral VF and contralateral LF from E3 chick NTs electroporated for 48 h (E3+24 hpe) with Tubb3::CAG:control, Tubb3::CAG:LRPdn or Tubb3::CAG:sβ-CatΔC. Each data point=6 slices for control, 9 for LRPdn or 8 for sβ-CatΔC. (F) Transverse sections of E2 chick NTs electroporated for 24 h (E2+24 hpe) with Ngn1 (nuclear GFP; green), membrane GFP (green) and Top::H2B·RFP (nuclear RFP; magenta). Nuclei are indicated by arrows and the apical processes by asterisks. Green arrows/asterisks show cells for which the nucleus is close to the apical border and have neural stem cell morphology, magenta arrows/asterisks indicate cells with the nucleus occupying basal positions but with the apical process still attached to the apical border, and blue arrows/asterisks indicate cells in which the apical abscission has already occurred. Scale bar: 10 μm. (G) Representative images of E3 chick NTs transfected for 24 h with Tubb3::Top:mGFP and cultured *ex vivo* for 950 min (E3+24 hpe+950 min). mGFP levels reflect Top activity in neurons. Scale bar: 10 μm. (H) Integrated GFP fluorescence density (IFD) of the cell shown in G calculated from flattened images of the *z*-planes containing the entire cell at each time point. Images were taken every 5 min. The shaded areas indicate the periods in which the apical abscission and neurite formation took place. (I) A similar experiment as in H, but following a cell in which the apical abscission had already started when the time lapse was initiated. Images were taken every 10 min. Scale bar: 10 μm. (J) Plot showing the GFP FI of the cell shown in I. (K) Schematic representation in grayscale of Top activity along neuron differentiation. Bar graphs show the mean±s.e.m. *P<0.05, ****P<0.0001; ns, non-significant (two-way ANOVA plus Dunnett's multiple comparisons test).

The canonical Wnt pathway is mediated by its Frizzled membrane receptors, a co-receptor called LRP5/6 and β-catenin, among others (Fig. 5A). We used the Tubb3:Cre system with a dominant-negative form of LRP5/6 (LRPdn; Fig. 5B) and a mutant β-catenin with diminished transcriptional activity (sβ-CatΔC; Fig. 5C) to study the effects of Wnt proteins and β-catenin on commissural neuron differentiation. Notably, we observed that cells expressing LRPdn extended axons normally, despite that the expression of this construct in E2 chick neural tubes clearly inhibited the expression of Top:H2B·RFP in both the dorsal Wnt response domain and the dorsal root ganglia (Fig. 5D, Fig. S3). By contrast, the number of axons from cells expressing sβ-CatΔC was significantly reduced in the commissure and the contralateral VF (Fig. 5E, Fig. S4A). These results indicated that DATT was mediated by β-catenin but not by Wnt proteins. To study the chronology of DATT, we transfected E2 chick embryos for 24 h with Ngn1 to induce differentiation, mGFP to show the cell contour and Top:H2B·RFP to monitor Tcf activity. Slices were then stained with anti-β-catenin and 4′,6-diamidino-2-phenylindole (DAPI) (Fig. 5F). We observed transfected dorsal cells at different stages of delamination: cells close to the apical border with NSC morphology (green arrows in Fig. 5F), cells at basal positions but still attached to the apical border (magenta arrows and asterisks in Fig. 5F) and cells already delaminated (blue arrows and asterisks in Fig. 5F). Top activity (nuclear accumulation of RFP) was higher in delaminated neurons; however, it was already elevated in basal pre-delaminated cells. Moreover, nuclear accumulation of endogenous β-catenin was evident in these two groups of cells. To better define the time sequence of Top activation during neural differentiation, we transfected E3 embryos for 12 h with Tubb3::Top:mGFP plus H2B·RFP and prepared *ex vivo* cultures from which we took time-lapse confocal images for a period of 950 min (Fig. 5G, Movie 1). We calculated the integrated GFP fluorescence density (IFD), which is shown as a percentage of the maximum (Fig. 5H). As suggested by fixed tissue experiments, the time-lapse experiments showed that Tcf-dependent transcription increased before apical abscission, then decreased at the beginning of apical foot retraction and subsequently increased again during neurite formation. In addition, we tracked cells that had already initiated the apical abscission at the beginning of the time-lapse acquisition to study Tcf transcription for as long as possible during axon growth (Fig. 5I, Movie 2). In this case, we observed that the increase in Top activity during neurite formation is also maintained during axon outgrowth and elongation (Fig. 5J). In Fig. 5K, we summarize in a schematic drawing the observed variation of Tcf-dependent transcription during neural delamination and axon elongation.

### β-catenin is redirected to the nucleus before apical foot abscission during dorsal neural differentiation

**Fig. 6.**
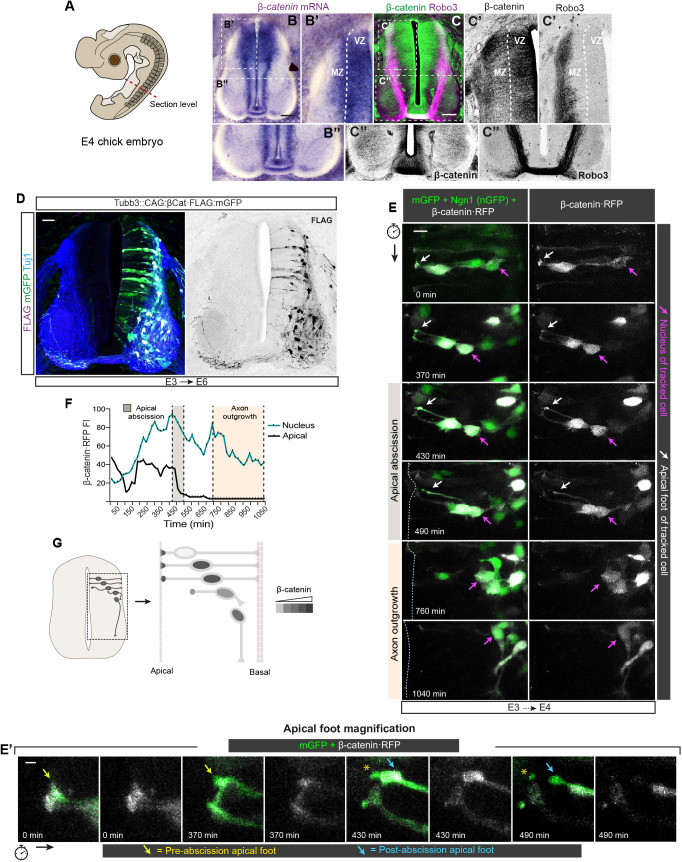
**β-catenin is redirected to the nucleus before apical foot abscission during neural differentiation.** (A) Drawing of an E4 chick embryo indicating the approximate thoracic level used to generate the transverse sections used in this work. (B) *In situ* hybridization showing *CTNNB1* mRNA distribution in transverse sections of E4 chick SC. (B′,B″) Enlargements of the areas labeled in B. Scale bar: 50 μm. (C) E4 SC sections stained with anti-β-catenin (green) and anti-Robo3 (magenta). (C′,C″) Enlargements of the areas labeled in C, with β-catenin and Robo3 staining shown separately in grayscale. Dashed lines in B′ and C′ represent the apical border. Scale bar: 50 μm. (D) E3 embryos electroporated for 72 h (E3+24 hpe) with Tubb3::CAG:sβ-Cat·FLAG:mGFP (express β-catenin·FLAG in neurons) stained with anti-FLAG (magenta) and anti-Tubb3 (Tuj1) (blue) antibodies. FLAG staining is shown in grayscale on the right. Scale bar: 50 μm. (E) Representative images of E2 chick NTs transfected for 24 h with mGFP (membrane; green), Ngn1 (nuclear GFP; green) and β-catenin·RFP (grayscale) and cultured *ex vivo* for 1040 min (E2+24 hpe+1040 min). RFP signal is shown in grayscale in the right column. In each time-lapse image, the position of the apical process and the nucleus of the tracked cell are indicated by white and magenta arrows, respectively. Scale bar:10 μm. (E′) Magnified images of mGFP (green) and β-catenin·RFP (grayscale) distribution in the apical foot during apical abscission. Pre- and post-abscission apical feet are indicated by yellow and blue arrows, respectively. The membrane stump left behind after abscission is indicated by yellow asterisks. Scale bar: 2 μm. (F) RFP FI measured in the apical foot (black line) and the nucleus (green line) at each time point. Images were taken every 10 min. The shaded areas indicate the periods in which the apical abscission and axon outgrowth took place. (G) Schematic representation of β-catenin·RFP distribution (in grayscale) along neuron differentiation.

The Wnt canonical pathway had been shown to collaborate in the early establishment of dorsal identities during NT development (Muroyama et al., 2002; Alvarez-Medina et al., 2008; Andrews et al., 2019). However, here we have demonstrated that Tcf-dependent transcription is both activated and required during commissural neurogenesis in a process that requires β-catenin but not Wnt proteins. Therefore, we used E5 sections (Fig. 6A) to study the distribution of β-catenin mRNA (Fig. 6B) and β-catenin protein (Fig. 6C). Interestingly, β-catenin mRNA was very abundant at the boundaries between the VZ and the MZ in the dorsal SC, in which different populations of interneurons are differentiating at this stage (Fig. 6B′). However, although β-catenin protein was mostly observed with N-cadherin at the AJs of the apical border along the SC, it was also present in most basal locations in the dorsal VZ and the cell bodies of the most dorsal Robo3-expressing neurons (Fig. 6C,C′). β-catenin protein was very abundant in the commissure, as was Robo3 (Fig. 6C″). In agreement, we observed that in Tubb3::CAG:βCat·FLAG embryos (electroporated at E3 for 24 h), β-catenin·FLAG protein was mainly accumulated in the nucleus and axons of neurons (Fig. 6D). During neurogenesis, differentiating neurons detach their apical process and retract it towards the cell body, leaving a stump where it had been attached (Das and Storey, 2014). Before detachment, N-cadherin levels decrease at the cellular apical pole (Rousso et al., 2012; Das and Storey, 2014). Therefore, we wondered whether DATT was fueled by the β-catenin released during AJ dismantling. We transfected E3 embryos with β-catenin·RFP, mGFP and Ngn1 for 12 h to prepare *ex vivo* cultures that were followed by confocal time lapses of the dorsal SC for an additional period of 1050 min (Fig. 6E,E′, Fig. S4B, Movies 3 and 4). Initially, β-catenin·RFP was more abundant in the AC than in the nucleus. Later, it steadily increased in the nucleus without decreasing in the AC, where it remained mostly constant until the apical abscission initiation. Notably, coincident with the results on Tcf-dependent transcription shown in Fig. 5, nuclear β-catenin·RFP reached its maximum before apical foot abscission, and although it progressively decreased later, it remained substantially elevated during axon outgrowth and elongation (Fig. 6F, Fig. S4C). In Fig. 6G, we summarize in a schematic drawing the β-catenin·RFP levels observed in the AC and the nucleus before and after apical foot abscission and during axon elongation. The results shown in Fig. 5 and Fig. 6 demonstrated that both Tcf-dependent transcription and nuclear β-catenin·RFP were raised before the AJs were dismantled. Therefore, we concluded that the rise in nuclear β-catenin, and consequently in Tcf-dependent transcription, depends on a neural-differentiation-prompted mechanism that either redirects newly produced β-catenin directly to the nucleus or interferes with the destruction complex, allowing its nuclear accumulation.

## DISCUSSION

### β-catenin/Tcf-dependent transcription is reactivated during SC commissural differentiation in a Wnt-independent manner

The Wnt canonical pathway (β-catenin/Tcf dependent) participates in defining dorsal fate and maintains stemness of neural progenitors, whereas its inhibition induces premature cell cycle exit and ventral patterning during early SC development (Alvarez-Medina et al., 2008; Wang et al., 2011; Herrera et al., 2014). However, we have now demonstrated that, later, during the first wave of neurogenesis, β-catenin/Tcf-dependent transcription is reactivated during neuronal differentiation of different subtypes of chick spinal commissural interneurons. Moreover, we have observed that this reactivation is an absolute requirement for the growth of pre-crossing commissural axons. Similarly, β-catenin/Tcf-dependent transcription is also activated early in the neural precursors of the mouse cortex VZ during radial glial cell expansion, and although it is silenced while these cells exit the VZ and migrate towards the cortical plate, it is later reactivated in a set of young neurons as they mature in the cortical plate (Woodhead et al., 2006). Although the involvement of Wnt proteins in the activation of β-catenin/Tcf-dependent transcription was not studied, the widespread distribution of this set of cortical neurons suggests that a Wnt factor gradient does not trigger this activation. In the present study, we demonstrated that, unlike Tcf repression, the expression of the dominant-negative form of the Wnt co-receptor LRP5/6 in Tubb3-expressing cells does not affect the outgrowth and pathfinding of spinal commissural axons. In addition, our experiments in which we overexpress Ngn1 or p27^Kip1^ show that the induction of premature differentiation correlates with an increase in nuclear localization of β-catenin and subsequent β-catenin/Tcf-dependent transcription in differentiating neurons. Although Wnt-dependent axon guidance had mainly been attributed to the PCP pathway, defects in post-crossing axons have been observed in knockdowns of LRP5/6 in chick neural tubes, indicating that the Wnt canonical pathway might also be participating after midline crossing (Avilés and Stoeckli, 2016). However, the activation of β-catenin/Tcf-dependent transcription in pre-crossing commissural neurons is likely to be an inherent consequence of the differentiation process rather than being influenced by external Wnt proteins.

### β-catenin translocation to the nucleus during commissural neuron differentiation

N-cadherin is responsible for the maintenance of the apical attachment (Wong et al., 2012), and the delamination process is associated with a downregulation of N-cadherin induced by pro-neuronal transcription factors such as neurogenins (Rousso et al., 2012; Das and Storey, 2014). Newly synthesized β-catenin has two possible outcomes: it can interact with N-cadherin at the endoplasmic reticulum and travel to the AJs, or it can be translocated into the nucleus. Downregulation of N-cadherin during neuron delamination could favor the transcriptional route. We have observed that both nuclear accumulation of β-catenin and DATT begin without a decrease in β-catenin at the AC. Thus, although the downregulation of N-cadherin could be involved, the AJs do not seem to be the primary source of the β-catenin that accumulates in the nucleus during commissural neuron differentiation. Our data show that DATT is activated during neuron delamination, continues to be active in pre-crossing commissural axons and eventually is turned down in post-crossing commissural axons. Therefore, it is most likely that different concurrent and sequential events regulate the nuclear accumulation of β-catenin during the process of differentiation. In the mouse cerebral cortex, the expansion of intermediate progenitors depends on the interaction of Axin with GSK3β in the cytoplasm. However, as neural differentiation begins, Axin is phosphorylated by β-catenin-associated Cdk5, causing the nuclear translocation of the Axin/β-catenin complexes and the activation of Tcf-dependent transcription. Interestingly, both nuclear accumulation of Axin/β-catenin and Tcf-dependent transcription are required to promote the differentiation of intermediate progenitors into neurons (Fang et al., 2013). Therefore, an equivalent mechanism might also be driving β-catenin to the nucleus during commissural neuron delamination. Slit interacts with Robo to induce the association of Robo/Abl complex with β-catenin/N-cadherin/Cables, causing the phosphorylation of β-catenin at Y489 by Abl that dissociates from N-cadherin and accumulates in the nucleus (Rhee et al., 2007). Thus, the induction of Robo1 and Robo2 expression by DATT might contribute to the maintenance of β-catenin/Tcf-dependent transcription in pre-crossing commissural neurons. A midline crossing mechanism that depends on the accumulation of β-catenin at the axon growth cone has recently been reported during midline crossing at the optic chiasm (Morenilla-Palao et al., 2020). In the suggested model, the differential expression of Wnt receptors would determine the effect of midline-expressed Wnt5a on the decision to cross the midline by commissural axons or to be repulsed in the case of ipsilateral axons. Although the expression of Fzd1, Fzd8 and LGR5 induced by Zic2 in ipsilateral neurons avoids the midline crossing by promoting a non-polarized distribution of β-catenin, other Wnt receptors such as Fzd3 have been proposed to respond to Wnt5a and mediate the required accumulation of β-catenin at commissural growth cones during midline crossing. Interestingly, our data have revealed accumulation of β-catenin in midline Robo3^+^ axons and positive regulation of Fzd7 and Fzd3 expression by DATT in spinal commissural neurons. The interaction of FP Wnt5a with some of these Wnt receptors might promote the accumulation of β-catenin at midline crossing growth cones and, therefore, the shutdown of the transcriptional signal in post-crossing axons. In summary, different mechanisms can operate from the initial steps of neural differentiation to the post-crossing stages in commissural neurons.

### The growth of pre-crossing commissural axons depends on genes activated by DATT during SC development

At the end of the 19th century, Ramón y Cajal proposed that FP cells secreted attractants for commissural axons that extend ventrally in the developing SC (Ramon y Cajal, 1911). To date, three FP-derived chemoattractive factors have been identified, netrin 1 (Kennedy et al., 1994), Shh (Charron et al., 2003) and vascular endothelial growth factor (VEGF) (Ruiz de Almodovar et al., 2011). However, only netrin 1 promoted the growth of axons and was required for the midline crossing (Moreno-Bravo et al., 2019; Wu et al., 2019). PANTHER pathway analysis informs that DATT positively regulates cell adhesion and axon guidance genes relevant during commissural neuron differentiation. Different receptors interact with netrin 1, such as DCC, UNC5C, Neo1 and Robo3 (Chédotal, 2019), and midline crossing by commissural axons is drastically reduced in mutant mice in which netrin 1, DCC or Robo3 are deleted (Serafini et al., 1996; Fazeli et al., 1997; Long et al., 2004). Although commissural axon crossing is normal in a severe hypomorphic Neo1 mouse model, it potentiates the effects caused by deleting DCC (Xu et al., 2014). The phenotype we observed after the suppression of Tcf-dependent transcription in chick SC is very similar to the one reported for DCC deletion, despite the fact that the *DCC* gene is lost in birds from the chicken family (Patthey et al., 2017). Interestingly, *NEO1* mRNA is expressed in developing chick SC in a pattern resembling that of mouse *Dcc* rather than *Neo1* mRNA, suggesting that, in chicken, the functions of DCC could have been assumed by Neo1 (Phan et al., 2011). Notably, *NEO1* was one of the genes for which expression was significantly downregulated after Tcf transcription inhibition. However, the Slit/Robo pathway is required for the correct axon guidance of post-crossing commissural axons through a repulsive mechanism exerted by Slit proteins secreted by the FP (Chédotal, 2019). In accordance, midline crossing is perturbed in Slit1, Slit2 and Slit3 triple knockouts (Long et al., 2004) or Robo1 and Robo2 double knockouts (Jaworski et al., 2010). Here, we have shown that DATT induces the expression of genes classified by PANTHER pathway analysis into the category called ‘axon guidance mediated by Slit/Robo’. Robo1 and Robo2 are expressed early in pre-crossing commissural axons; however, Robo3 increasingly reduces their presence in the growth cones of these commissural axons as they approach the midline to allow its crossing, being re-established after crossing to avoid re-entering. Although much attention has been devoted to the repulsion effect that midline-located Slit produces on post-crossing axons that express Robo1 and Robo2, the lack of motor column avoidance by the descending commissural axons in Slit and Robo knockout mice demonstrates a function of the Slit/Robo system in pre-crossing axon guidance (Long et al., 2004; Jaworski et al., 2010). Remarkably, in Hamburger–Hamilton stage (HH)16-17 chick SC (equivalent to E9.5 in mice), in which most interneurons are still initiating the axon extension process, *ROBO1* and *SLIT2* mRNAs are abundant in the areas occupied by the cell bodies of dorsal interneurons and the motor neurons, respectively (Mambetisaeva et al., 2005). In addition to its function in axon guidance, Robo1 has been demonstrated to control the balance between direct and indirect neurogenesis in the differentiation of the cerebral cortex (Cárdenas et al., 2018). The PANTHER pathway analysis showed the cadherin signaling pathway as the pathway with the second-highest combined score. Interestingly, N-cadherin dysregulation alters commissural projections in the SC; its overexpression markedly affected the navigation of post-crossing commissural axons, and its knockdown significantly reduced the presence of commissural axons (Yang et al., 2016). Our *in silico* analysis predicted *TCF7L2* as one of the main transcription factor genes regulating the aforementioned PANTHER pathways. In our study, *TCF7L2* knockdown entirely reproduced the phenotype induced by the suppression of Tcf-dependent transcription. Similarly, the pathfinding defects of the thalamocortical axons of forebrain-specific *Tcf7l2*-deficient mice were attributed to the downregulation of cell adhesion and axon guidance genes during the thalamic and habenular neuron differentiation (Lee et al., 2017; Lipiec et al., 2020). In addition, the *Neurod6:Ctnnb1^Ex3^* mouse, which expressed a stabilized form of β-catenin during the differentiation of cortical excitatory neurons, presented as the main phenotype a lack of hippocampal commissure (Morgan-Smith et al., 2014). In summary, our data and the abovementioned studies indicate that the genes regulated by β-catenin/TCF7L2 play important roles in axon pathfinding during neuronal differentiation.

## MATERIALS AND METHODS

### Commercial antibodies and chemicals

The following antibodies and reagents were used (the concentration indicated is the one used for immunohistochemistry): mouse antibodies against β-catenin (Sigma-Aldrich, C7207; 1:200) or Tubb3 (Covance, MMS-435P; 1:5000); rabbit antibodies against Sox2 (Invitrogen, 48-1400; 1:500) or RFP (our laboratory, Rb2; 1:5000); goat antibody against Robo3 (R&D Systems, AF3076; 1:500); Rhodamine–Phalloidin (Invitrogen, R415; 1:250).

### DNA constructs

#### Protein expression

pCIG (CMV-IE enhancer/b-actin promoter-IRES-nuclear GFP); pCS2-mRFP (CMV-IE94 promoter membrane RFP); Tubb3:Cre (TUBB3 enhancer/TK minimal promoter-CRE); CAG:LoxP·mGFP (b-actin promoter/CMV-IE enhancer-Lox-PolyA-Lox-IRES-mGFP); Top:LoxP·mGFP (5xWnt response element/TK minimal promoter-Lox-PolyA-Lox-IRES-mGFP); CAG:LoxP·TcfEnR (fusion of engrailed repressor to Tcf3 HMG box, cloned in CAG:LoxP·mGFP); CAG:LoxP·sβ-Cat·mGFP (β-catenin^S33Y^ cloned in CAG:LoxP·mGFP); CAG:LoxP·LRPdn (LRP6 with a C-terminus deletion cloned in CAG:LoxP·mGFP); CAG:LoxP· sβ-CatΔC (β-catenin^S33Y^ with a C-terminus deletion cloned in CAG:LoxP·mGFP); TopFlash (5xWnt response element/TK minimal promoter luciferase); Top:mGFP (5xWnt response element/TK minimal promoter membrane-GFP); Top:dGFP (5xWnt response element/TK minimal promoter dGFP); Top:H2B·RFP (5xWnt response element/TK minimal promoter histone2b·RFP).

#### shRNAs

pSHIN (H1 promoter for shRNAs plus SRα promoter for EGFP) was used to clone the following shRNAs: shScrambled, 5′-CCGGTCTCGACGGTCGAGT-3′ (control shRNA); shLEF1, 5′-CCCAGAACATCCAACAAGG-3′; shTCF7, 5′-CGCGGGACAACTACGGAAA-3′; shTCF7L1, 5′-ATGGGCGATGAAGCCAGGA-3′; shTCF7L2, 5′-TGAGCACTTCACACCAGGA-3′; shFOXM1, 5′-GCATCAACTCCTACCTTGT-3′.

### Affymetrix GeneChip arrays

Plasmid DNA encoding Tubb3:Cre and either CAG:LoxP·mGFP or CAG:LoxP·TcfEnR were co-electroporated into E3 chicken embryos for 24 h to produce either Tubb3::CAG:TcfEnR (suppression of Tcf-dependent transcription in neurons) or Tubb3::CAG:control (controls). Then, GFP^+^ neural tubes were dissected from the embryos under a fluorescence dissection stereomicroscope (Leica). For each experimental replicate, 20-25 dissected NTs of the same conditions were pooled, and a single-cell suspension was obtained following 10-15 min incubation with trypsin-EDTA (Sigma-Aldrich). GFP fluorescent cells were sorted using an FACS Aria III cell sorter (BD Biosciences). After cell sorting, total RNA was extracted by an RNeasy Micro Kit (Qiagen, 74004) according to the manufacturer's instructions. The quality and quantity of purified RNA were verified by Agilent Bioanalyzer 2100, and, subsequently, the RNA was hybridized and processed in accordance with the manufacturer's instructions (Affymetrix GeneChip Chicken Genome Array). The data were processed with the RMA algorithm. The data from three biological replicates of each experiment were averaged, and differentially expressed genes were selected. The results were filtered using thresholds of (log2 FC) ≥2 (TcfEnR-upregulated genes) or (log2 FC) ≤−2 (TcfEnR-downregulated genes) and *q*-value (adjusted *P*-value) ≤0.05. Affymettrix data are in GEO, accession number GSE234518.

### Chick embryo *in ovo* electroporation

Eggs from White-Leghorn chickens were incubated at 37.8°C in an atmosphere of 45% humidity, and the embryos were staged according to Hamburger and Hamilton (1992). Chick embryos were electroporated with column-purified plasmid DNA in H_2_O containing Fast Green (Merck, F7252-5G; 0.5 µg/µl). Briefly, plasmid DNA was injected into the lumen of HH12 (E2) or HH18 (E3) NTs, electrodes were placed on either side of the NT, and electroporation was carried out by applying five 50 ms square pulses using an Intracel Dual Pulse (TSS10) electroporator set at 25 V. Transfected embryos were allowed to develop to the specific stages and then dissected under a fluorescence dissecting microscope (Leica). In our conditions, HH12 embryos electroporated for 24, 36 and 48 h typically reached stages HH18, HH21 and HH23, respectively; HH18 embryos electroporated for 24 h and 24 h reached stages HH23 and HH26, respectively. Embryos that did not develop to the expected stages were discarded. To stage the different embryos we used the staging guide published in https://embryology.med.unsw.edu.au/embryology/index.php/Hamburger_Hamilton_Stages.

### Immunostaining and confocal fluorescence microscopy

Embryos were fixed overnight at 4°C in 4% PFA (4% paraformaldehyde in PBS) and then sectioned at 60 μm thickness with a vibratome (Leica, VT1000S). Immunostaining was performed following standard protocols. The entire procedure was carried out using PBT-BSA (PBS containing 0.1% Triton X-100, 0.1% bovine serum albumin and 0.05% sodium azide) using a gently rocking platform. Sections were pre-washed for 30 min at room temperature (RT), incubated overnight at 4°C with primary antibodies, washed three times for 20 min, incubated for 2 h at RT with Alexa Fluor- or cyanine-conjugated secondary antibodies, washed three times, and mounted with Fluoromount (Sigma-Aldrich). Slices were examined at 18°C on a Leica SP5 (20×/0.7 NA, 40×/1.25 1.25 objective) controlled under the Leica LAS software or a Zeiss Lsm 780 (25×/0.57 NA, 40×/1.3 NA, 63×/0.18 NA objective) multiphoton microscope controlled under the LSM Software ZEN 2.1. Images were manipulated using ImageJ software.

### *Ex vivo* chick NT slice culture and time-lapse imaging

Embryos were electroporated at stage HH18 (E3). After 24 h of incubation, GFP transfected areas were dissected in ice-cold L15 medium (Sigma-Aldrich, L4386) and embedded in 3% low-gelling-temperature agarose dissolved in neurobasal medium (Invitrogen) to be later sectioned at 250 μm thickness with a vibratome (Leica, VT1000S). Next, selected sections were placed on a 35 mm glass-bottom Petri dish (ibidi) and embedded in 0.5% low-gelling-temperature agarose dissolved in neurobasal medium. Slices were cultured in 2 ml neurobasal medium supplemented with B-27 (Thermo Fisher Scientific, 17504044) containing 2 mM L-glutamine. For time-lapse imaging, images were acquired every 5 min (160 *z*-planes spaced 0.93 µm, images of 1565×1024 pixels) with a Dragonfly 500 (Oxford Instruments) using a 20× objective (0.75 NA) (Fig. 5G, Movie 1) or every 10 min (20-25 *z*-planes spaced 1.5 µm, images of 1024×1024 pixels) with a Zeiss LSM780 confocal microscope using a 25× objective (0.80 NA) (Fig. 5I, Fig. 6E, Fig. S3B, Movies 2-4). The culture was maintained at 37.5°C in a humid atmosphere (95% air, 5% CO_2_). Images were processed using ImageJ/Fiji software.

### Whole-mount immunolabeling and optical clearing

HH18 (E3) chick embryos were electroporated for 24 h (E3+24 hpe) with pCS2-mRFP plus pSHIN-shTCF7L2 or pSHIN-shControl. Embryos were collected in ice-cold PBS, blood was drained out, and extraembryonic membranes were removed. Clean embryos were fixed overnight in 4% PFA at 4°C in a rotating wheel and for 1 h at RT, and finally washed three times for 1 h each with PBS. Whole-mount immunolabeling was carried out with modification of the iDISCO protocol (Renier et al., 2014).

#### Pre-treatment

Embryos were dehydrated through a freshly prepared methanol/PBS series (25%, 50%, 75% and finally 100% twice), 1 h each at RT. Next, embryos were bleached overnight at 4°C with ice-cold methanol containing 5% H_2_O_2_ and 20% dimethyl sulfoxide (DMSO). After that, embryos were washed at RT three times in methanol for 1 h each, once in methanol containing 20% DMSO for 2 h, and rehydrated in a freshly prepared methanol/PBS series (75%, 50%, 25% and 0%), 1 h each at RT. Finally embryos were washed twice with PBS containing 0.2% Triton X-100 for 1 h each at RT before immunostaining.

#### Immunostaining

Pre-treated embryos were incubated in PBS containing 0.2% Triton X-100, 20% DMSO and 0.3 M glycine overnight at 37°C in a shaker at 80 rpm. The next day, embryos were blocked with PBS containing 0.2% Triton X-100, 10% DMSO and 6% fetal bovine serum (FBS) at 37°C in a shaker at 80 rpm for 1 day. Then, embryos were washed twice in PBS containing 0.2% Tween 20 and 10 µg/ml heparin (PTwH) for 1 h each. After that, embryos were incubated with primary antibodies overnight at 37°C and 80 rpm, in PTwH containing 5% DMSO and 3% FBS (goat anti-Robo3 at 1:300 and rabbit anti-RFP at 1:300). The next day, embryos were washed in PTwH for 10 min, 15 min, 30 min, 1 h, 2 h and 4 h, and then incubated with the secondary antibodies in PTwH contain 3% FBS overnight at 37°C and 80 rpm [anti-rabbit Alexa Fluor 555 (Thermo Fisher Scientific, A-21428) and anti-goat Alexa Fluor 647 (Thermo Fisher Scientific, A-21447) at 1:300]. Finally embryos were washed in PTwH for 10 min, 15 min, 30 min, 1 h, 2 h and 4 h stored at 4°C in PBS before clearing.

#### Optical clearing

After washing with PBS for 30 min, chick embryos were embedded into a 0.8% solution of low-melting agarose (Sigma-Aldrich, A9045) diluted in MilliQ water to enable easy mounting of the sample in our custom light-sheet system. We used a 5 ml syringe as a mold for that. For refractive index (RI) matching between the sample and the embedding agarose, the blocks containing the tissue were first dehydrated with three incubation changes of methanol 100% at 4°C and finally immersed in a 1:2 mixture of benzyl alcohol (Sigma-Aldrich, 2412-1l) and benzyl benzoate (Sigma-Aldrich, W213802-1KG-W) (BABB), at RT for 2-3 days before imaging.

### Light-sheet fluorescence microscopy

Once the RI was matched, 3D imaging of the entire tissue was carried out using light-sheet fluorescence microscopy. For the mesoscopic analysis of the embryos, light-sheet fluorescence macroscopy based on a custom instrument (MacroSPIM) was used (Kennel et al., 2018). In brief, the agarose block containing the embryo was placed vertically on an underlying rotation platform inside a 2 mm wall quartz cuvette and immersed in BABB. Imaging was performed horizontally with a AZ100 M macroscope lens system (Nikon) at a magnifications of 9.6×, yielding respective pixel size *xy*=0.677 µm, with *z*-steps of 2.5 µm. The light-sheet waist was adjusted to yield ∼4.5-5 µm axial resolution. Fluorescence of Alexa Fluor 555 was excited with a 561 nm DPSS laser and collected with a ‘brightline’ filter BP609/54 (Semrock), and fluorescence of Alexa Fluor 647 was induced with a 638 nm solid-state laser, collected with a ‘Edge basic’ 635 LP (Semrock). Images were captured by a Flash4.0 v2 SCMOS camera (Hamamatsu), and 3D reconstruction images were generated using ImageJ/Fiji software.

### Chick embryonic fibroblast (CEF) cultures and electroporation

CEFS were obtained from chick embryos at HH30-35 (7-9 days of development). The embryos were mechanically disaggregated with forceps and then treated with trypsin-EDTA for 15 min at 37°C. DNAse I (final concentration 100 U/ml) was added to digest the DNA resulting from cell damage, and trypsin was inactivated by adding one volume of culture medium with 10% FBS. The cell suspension was centrifuged for 5 min at 300 ***g*** to eliminate aggregates, and the supernatant was resuspended in Opti-MEM™ (Thermo Fisher Scientific, 31985062) at the desired cell concentration. For electroporation, CEFs were resuspended at 15,000,000 cells/ml. Cell cultures were maintained in an atmosphere of 5% CO_2_ at 37°C.

Transient transfection of CEFs was carried out with an electroporator (Microporator MP 100, Digital Bio) applying a pulse of 1200-1400 V for 20 ms. Transfected cells were seeded according to the experiment to be carried out. For RT-qPCR experiments, 1.5-3 million cells per well were seeded in six-well culture plates and grown for 24 h.

### *In situ* hybridization

HH18 chick embryos were fixed overnight at 4°C with 4% PFA. The next day, embryos were dehydrated with increasing concentrations of methanol (25%, 50%, 75% and 100%) in PBT buffer (PBS containing 0.1% Tween 20) until performing the procedure. Whole-mount *in situ* hybridization was performed following standard procedures. To detect chick *CTNNB1* mRNA, the following 1046 bp probes were used: 1, 5′-CGAGACAGCGGATCTTGGACTTGACATTGGTGCCCAGGGAGAACCTCTTGGATACCGCCC-3′; 61, 5′-AGATGATCCTAGCTACCGTTCTTTCCACTCTGGCGGATACGGTCAGGATGCCTTGGGTAT-3′; 121, 5′-GGACCCTATGATGGAACATGAAATGGGTGGCCACCACCCTGGTGCTGACTACCCAGTTGA-3′; 181, 5′-TGGTCTGCCAGATCTTGGCCATGCCCAGGACCTTATGGATGGGCTGCCTCCAGGTGACAG-3′; 241, 5′-TAATCAGTTGGCCTGGTTCGATACTGACCTGTAAATCATCCTTTAGCTGTATCATCTGAA-3′; 301, 5′-TGAACTTGCATTGATTGGCCTGTAGAGTTGCTGAGAGGGCTCGAGGGGTGGGCTAGTATC-3′; 361, 5′-TCAGAAAGTGCCTGACACACTAACCAAGCTGAGTTTCCTATGGGAACAATTGAAGTAAAC-3′; 421, 5′-TTTTTGTTCTGGTCCTTTTTGGTCGAGGAGTAATAATACAAATGGATTTTGGGAGTGATT-3′; 481, 5′-CAAGAAACGAGGAATGCACAAGAATGAATTGCAAGATGGAATTTATCAAACCCTAGCCTT-3′; 541, 5′-GCTTGTTAAAAATTTATTATTTTTTTTAAATCTCTGTAATGGTACTGACCTTTGCTTGCT-3′; 601, 5′-TTGAAAGTAGCCTTTCTTTTCGCAGTAATTGTTGTTAGGTTTTTTTTTTTAAGTCTCTCG-3′; 661, 5′-TAGTATTAAGTTATAGTGAATATGCTACAGCAGTTTCTAATTTTTAAGGATTGAGTAAAG-3′; 721, 5′-GTGTAGAACACTAATTCATAATCGCTCTAACTGTATTCTGAATAAAGTGTAACATTGTGT-3′; 781, 5′-AGCCTTTTTGTATAAAAAAACTAGACAAATAGAAATGGTCCAATTAGTTTCCTTTTTAAT-3′; 841, 5′-ATGCTTAAAATAAGCAGGTGGATCTATTTCATGTTTTTGATCAAAAACTTTATCTGGGAT-3′; 901, 5′-ATGTCTGGGTAGGGGCCAGTAAGAACTGTTTATTTGGAACCTTGTATTGGACAGTTTACC-3′; 961, 5′-AGTTGCCTTTTATCCCAAAGTTATTGTAGCCTGCTGTGATACAGATGCTTCATGAGAAAA-3′; 1021, 5′-ATGCAGTTATAAAATGGTTCAAAATT-3′.

The probes to detect chick *LEF1*, *TCF7*, *TCF2L1* and *TCF7L2* mRNAs were generously provided by Dr Elisa Marti (Alvarez-Medina et al., 2008). Hybridized embryos were post-fixed in 4% PFA, embedded in 5% agarose–10% sucrose blocks and then sectioned with a vibratome (Leica, VT1000S) at 60 μm thickness. Sections were photographed with an Olympus DP72 digital color camera attached to a Nikon E600 microscope. The data show a representative image obtained from three embryos.

### *In vivo* luciferase reporter assay

Embryos were electroporated with the DNAs indicated together with a 5xTcf-BS luciferase reporter construct containing synthetic Tcf-binding sites (TopFlash), as well as with a Renilla construct (Promega) for normalization. GFP-positive NTs were dissected at 48 hpe and homogenized in Passive Lysis Buffer (Promega, E1910). Firefly and Renilla luciferase activity was measured by the Dual Luciferase Reporter Assay System (Promega).

### Immunofluorescence intensity quantification in fixed tissue slices

Five or six successfully transfected embryos were always used to generate the slices used for quantification. To avoid bias during image acquisition for parameter quantification, we implemented a coding system that concealed the true identity of each image until after analysis. The images were acquired using the same gain and laser parameters. In order to compare the fluorescence intensity in the axons of different compartments, specifically funiculi and commissure, across different treatments, and to make it independent of the transfection level, it was crucial to reference it to the fluorescence measured in the somas of the neurons that emitted the axons. To define the soma regions, both Robo3 and Tubb3 staining were utilized. For each area of interest, the area, integrated density and mean gray value were measured. Three neighboring selections with no fluorescence were also measured for background readings. The areas of interest were delineated using the freehand line tool in ImageJ/Fiji, with Robo3 or Tubb3 staining as references. To normalize the intensity of GFP fluorescence in the axons, the following formula was used:

normalized GFP intensity in axons=(GFP intensity in axons within the region of interest−mean fluorescence of background readings)/(GFP intensity in somas within Robo3^+^ or Tubb3^+^ region−mean fluorescence of background readings).

First, the mean GFP intensity in the axons within the region of interest (ROI) was measured, with the average background fluorescence subtracted. Then, the mean GFP intensity in the soma of commissural cells (within the Robo3^+^ or Tubb3^+^ region) was calculated, also with the average background fluorescence subtracted. Finally, the first value was divided by the second value to obtain the normalized GFP intensity in the axons.

### Fluorescence intensity quantification in *ex vivo* time-lapse imaging

For Top-GFP fluorescence quantification (Fig. 5H,J), the *z*-planes containing the entire studied cell were manually selected and flattened into a single 2D image per each time point. An ROI was defined manually for the entire time sequence and the background was subtracted with ImageJ built-in rolling ball algorithm using a rolling ball radius of 25 pixels (0.34 µm/pixel). The IFD of the ROI was measured with ImageJ and represented as the percentage of the maximum. For β-catenin·RFP fluorescence quantification (Fig. 6F, Fig. S4C), ROIs containing the apical pole and the nucleus were manually defined for each time point. The mean fluorescence intensity of the mentioned ROIs and three neighboring selections with no fluorescence (background measurements) were calculated with ImageJ for the β-catenin·RFP channel at each time point. β-catenin·RFP fluorescence intensity was generated by calculating the mean β-catenin·RFP intensity within the ROI after subtracting the background.

### Statistical analyses

GraphPad Prism 6 software was used for statistical analysis. A normality study of the data was carried out in all experiments through the D’Agostino–Pearson test. For statistical analysis, different statistical tests were used depending on the nature of the data to be compared, as suggested by GraphPad Prism built-in assistance. These are indicated in individual figure legends (unpaired two-tailed *t*-test was used in Fig. S2C).

Quantitative data with normal distribution are expressed as the mean±s.e.m. In all cases, a degree of significance was established with a *P*-value <0.05. A confidence limit greater than 95% was established.

## Supplementary Material

10.1242/develop.201651_sup1 Supplementary information
