# Supplementary material for: Neurogenesis redirects β-catenin from adherens junctions to the nucleus to promote axonal growth
Source: Development. 2023 Aug 24;150(16):dev201651. doi: 10.1242/dev.201651 (PMC10482005; doi:10.1242/dev.201651)
Supplement: Supplementary information [file develop-150-201651-s1.pdf]

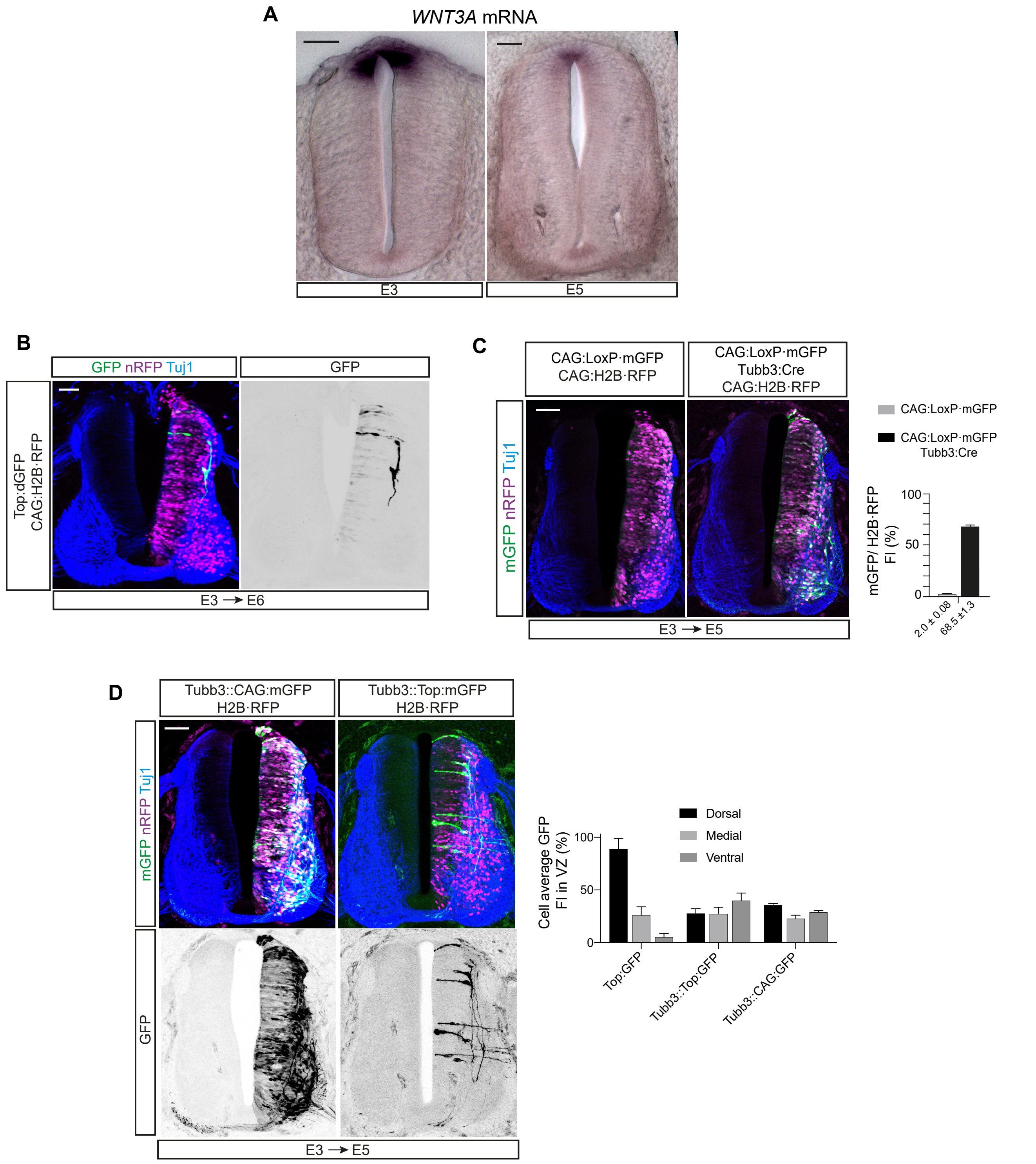

Fig. S1. (A) In situ hybridization showing *WNT3A* mRNA distribution in transverse sections of E3 and E5 chick SC. Scale bar = 50 µm. (B) E3 chick NTs electroporated for 72 h (E3 + 24 h) with Top:dGFP (destabilized GFP, green) and CAG:H2B·RFP (nuclear RFP, magenta). Transverse SC slices were stained with Tuj1 antibody (blue). dGFP is shown alone in greyscale at the right-handed panel. (C) E3 chick NTs electroporated for 48 h (E3 + 24 h) with CAG:LoxP·GFP plus CAG:H2B·RFP without (left panel) or with (right panel) Tubb3:Cre. The bar-graph shows the ratio of fluorescence intensity between mGFP and H2BRFP in ventricular zone (D) E3 chick NTs electroporated for 48 h (E3 + 24 h) with Tubb3::CAG:mGFP (shows all transfected neurons, membrane, green) or Tubb3::Top:mGFP (shows the Tcf/Lef activity in transfected neurons, membrane, green) and CAG:H2B·RFP (shows nucleus, magenta). E5 transverse SC slices were stained with anti Tuj1 antibody (stains all neurons, blue). The bar-graph shows the mean GFP fluorescence intensity in the dorsal, medial and ventral ventricular zone in neural tubes with the indicated transfections.

Development • Supplementary information

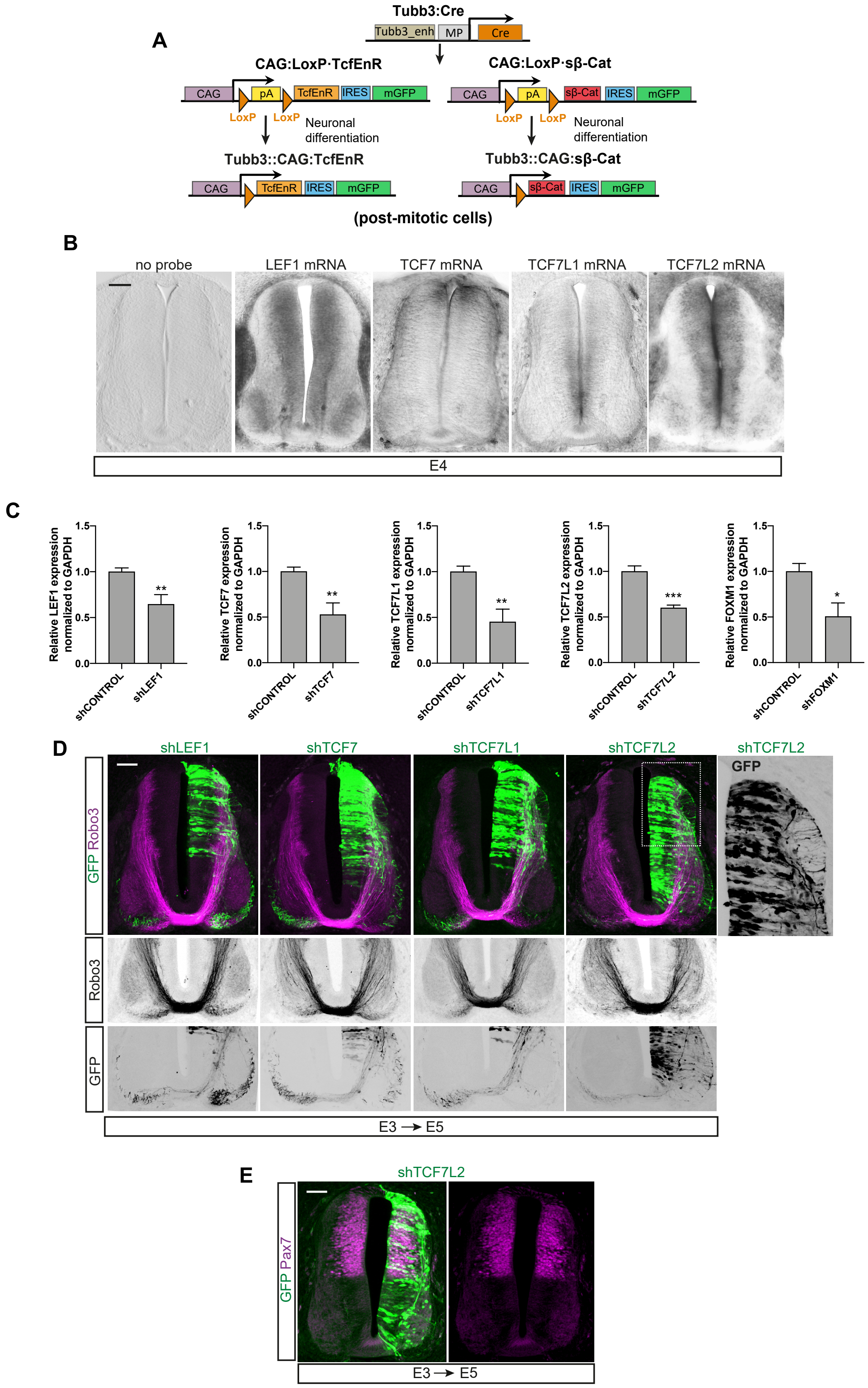

Development • Supplementary information

Fig. S2. (A) Scheme of vectors used to generate Tubb3::CAG:TcfEnR and Tubb3::CAG:sβ-Cat embryos. (B) In situ hybridization showing in grey scale LEF1, TCF7, TCF7L1 or TCF7L2 mRNA distribution in transverse sections of E4 chick SC. (C) Bar-graph showing the efficiency of the different short-hairpin inhibitory RNAs (shRNAs) designed against the different Tcf/Lef transcription factors expressed in chick NTs and FOXM1. The shRNAs were cloned in the GFP expressing vector pSHIN and tested in chick embryonic fibroblast cultures by RT qPCR. (D) E3 chick NTs electroporated for 48 h (E3 + 24 h) with GFP-expressing pSHIN vectors encoding shRNAs against LEF1, TCF7, TCF7L1, or TCF7L2. SC transverse sections were stained with anti-Robo 3 antibody (magenta), and GFP (green) indicates transfection. The content of the area delimited by a dotted line in the shTCFL2 panel is magnified at the right-handed panel showing in greyscale the GFP channel. (E) E3 chick NTs electroporated for 48 h (E3 + 24 h) with an shRNA targeting TCF7L2 (cloned in pSHIN vector), SC transverse sections were stained with anti Pax7 antibody (an interneuron marker, magenta), GFP (green) indicates transfection.

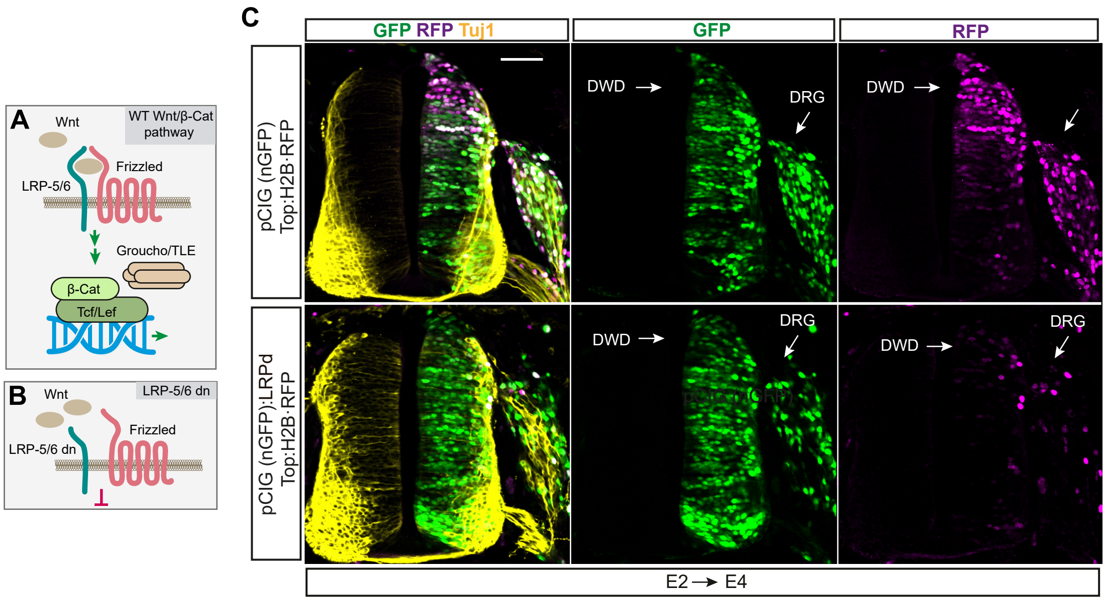

Fig. S3. (A) Simplified scheme illustrating wild-type canonical Wnt pathway where binding of Wnt proteins to Frizzled receptor and its co-receptor LRP 5/6 activates Tcf dependent transcription. (B) The pathway cannot be initiated at receptor level in the presence of a dominant negative form of LRP 5/6 with a C-terminal deletion. (C) E2 chick NTs electroporated for 40 h (E2 + 40 h) with TOP-H2B·RFP (expresses nuclear RFP under a Wnt response element, magenta) plus pCIG-LRPdn (expresses the dominant negative form of LRP5-6 and nuclear GFP as a bicistronic element) or pCIG (green). SC transverse sections were stained with Tuj1 antibody (yellow). In this case we observed that pCIG-LRPdn expression clearly inhibited the expression of TOP-H2B·RFP in the dorsal Wnt response domain and the dorsal root ganglia (DRG), two regions in which endogenous Wnt factors activate Wnt signalling in E2-E4 chick neural tubes.

Development • Supplementary information

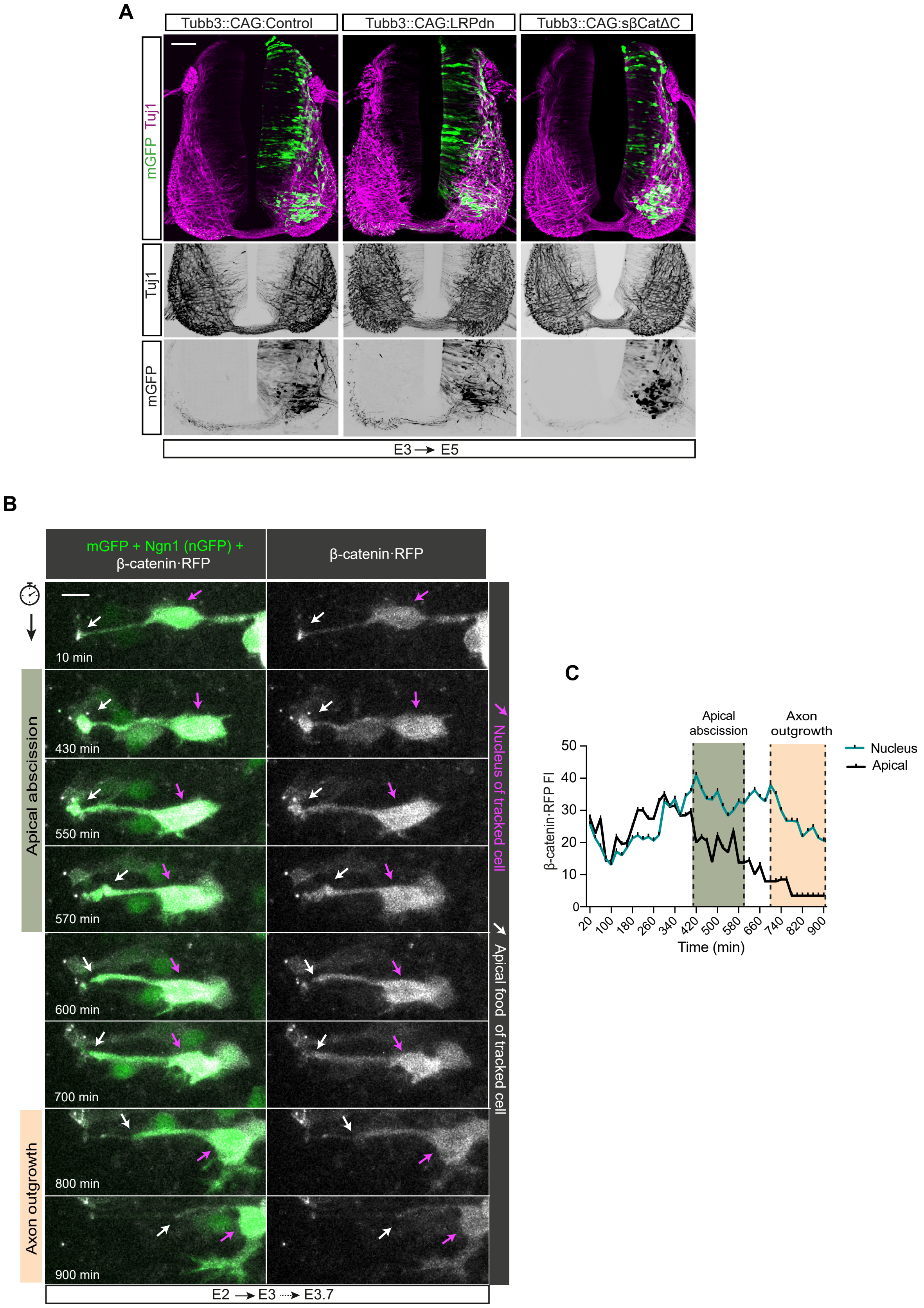

Fig. S4. (A) E3 chick NTs electroporated for 48 h (E3 + 24 h) with Tubb3::CAG:control, Tubb3::CAG:LRPdn or Tubb3::CAG:sβ-CatΔC. Transverse SC slices were stained with Tuj1 antibody (magenta). (B) Representative images of E2 chick NTs transfected for 24 h with mGFP (green, membrane), Ngn1 (green, nuclear GFP indicates transfection), and β-catenin·RFP (greyscale) and cultured ex vivo for 900 min (E2 + 24 h + 900 min). RFP signal is shown alone in greyscale in the right-handed panels. In each time-lapse, the position of the apical process and the nucleus of the tracked cell are indicated by white and magenta arrows, respectively. (C) The plot shows the RFP fluorescence intensity measured in the apical foot (black line) and the nucleus (green line) over time. Images were taken every 10 min. The shaded areas indicate the periods in which the apical abscission and axon outgrowth took place.

Table S1. Tubb3 dependent gene expression. The table contains three separated columns enumerating all the genes processed in the array, the genes that are upregulated and the genes that are downregulated.

Click here to download Table S1

Table S2. Tubb3 dependent gene expression meta data. The table contains the metadata of the Afimetrix gene expression study.

Click here to download Table S2

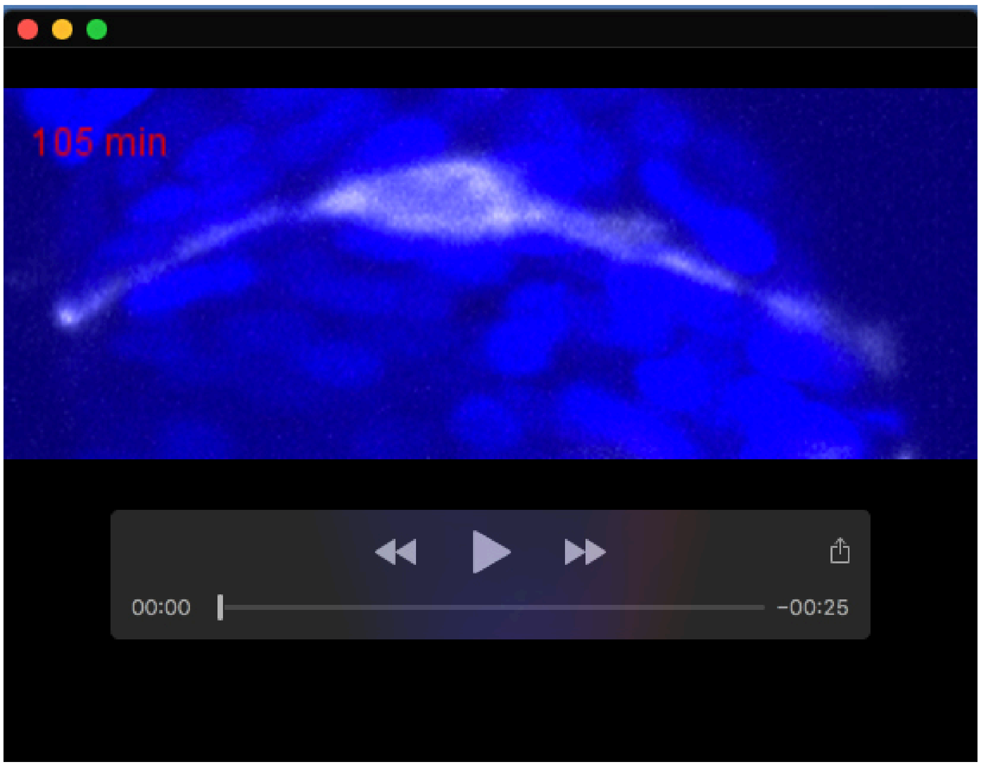

Movie 1. E3 chick NTs transfected for 24 h with Tubb3::Top:mGFP plus CAG:H2b·RFP and cultured ex vivo for 950 min (E3 + 24 h + 950 min). Images were taken every 5 min. mGFP (grey scale) levels reflects Top activity in neurons, nuclear RFP (blue) indicates transfection.

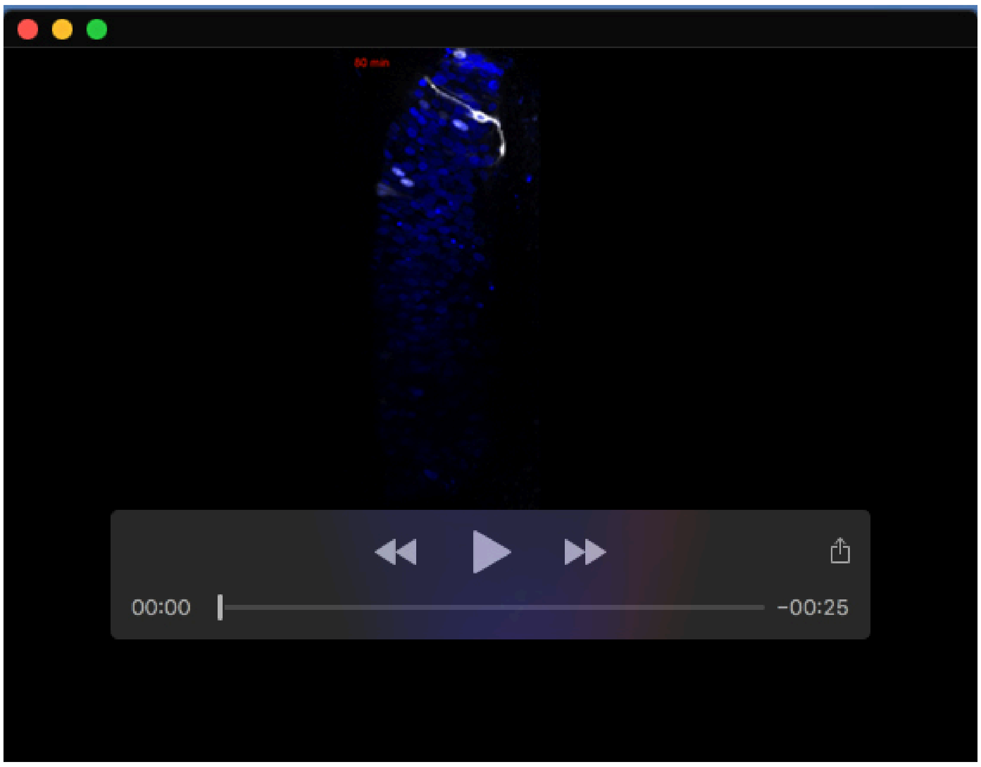

Movie 2. E3 chick NTs transfected for 24 h with Tubb3::Top:mGFP plus CAG:H2b·RFP and cultured ex vivo for 800 min (E3 + 24 h + 800 min). Images were taken every 10 min. mGFP levels reflect Top activity in neurons, nuclear RFP (blue) indicates transfection.

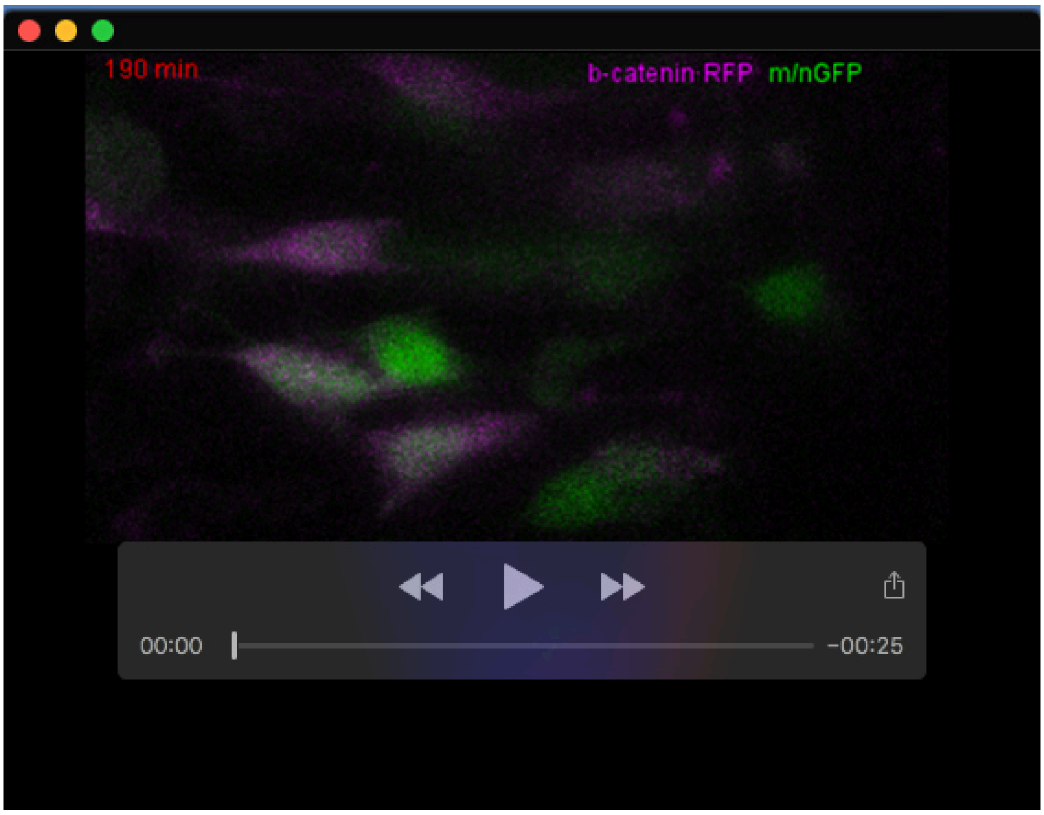

Movie 3. E2 chick NTs transfected for 24 h with mGFP (membrane GFP, green), Ngn1 (nuclear GFP, green) and β-catenin·RFP (magenta) and cultured ex vivo for 1040 min (E2 + 24 h + 1040 min). Following two channel images, β-catenin·RFP channel is shown separately in greyscale. Images were taken every 10 min.

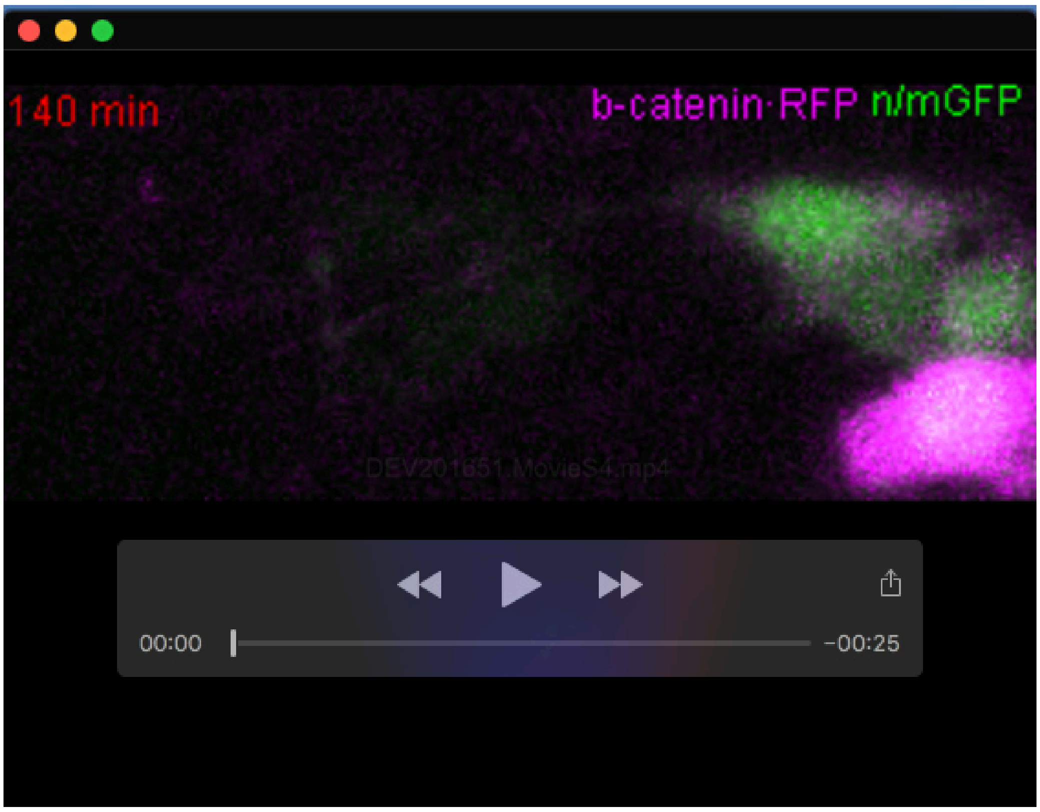

Movie 4. E2 chick NTs transfected for 24 h with mGFP (membrane GFP, green), Ngn1 (nuclear GFP, green), and β-catenin·RFP (magenta) and cultured ex vivo for 900 min (E2 + 24 h + 900 min). Following two channel images, β-catenin·RFP channel is shown separately in greyscale. Images were taken every 10 min.

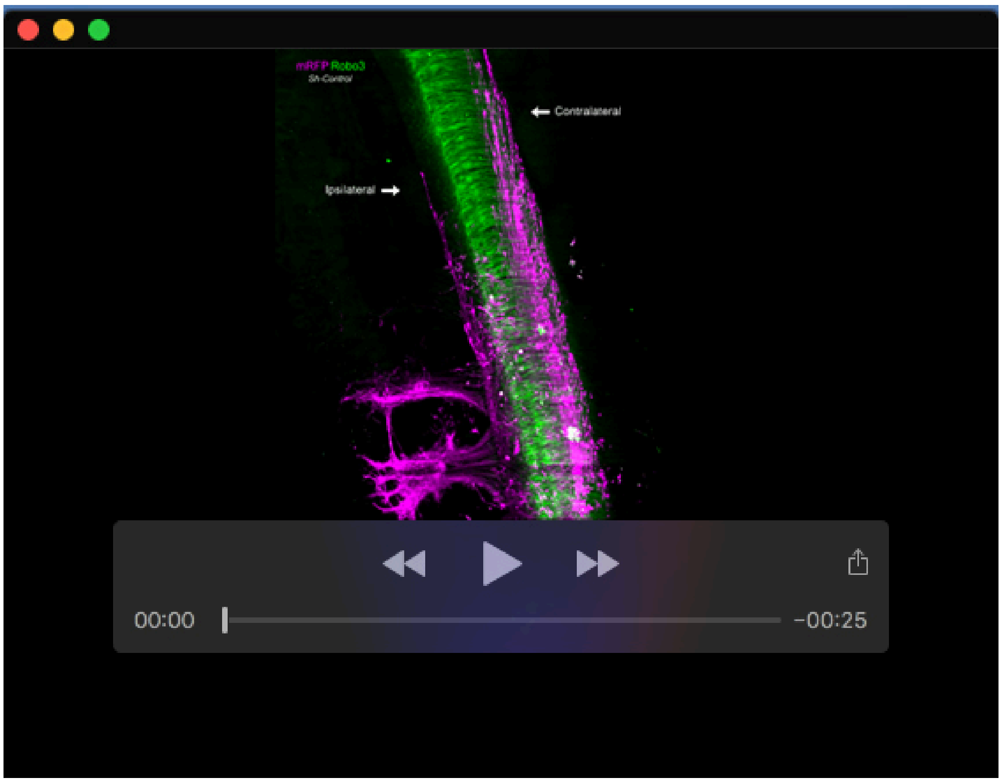

Movie 5. 360º view of 3D reconstruction of volume imaging performed through light sheet fluorescence microscopy at 9.6x of cleared E3 chick embryos electroporated for 24 h (E3 + 24 hpe) with pCS2-mRFP plus pSHIN-ShControl that were whole-mount immuno labeled with anti RFP (magenta) and anti Robo3 (green) antibodies. The RFP channel is shown alone in grey scale after the magenta/green sequence. The image shows 1º steps of a 360º rotation with the axis placed in the cranial-caudal direction. 3D reconstruction was generated with ImageJ/Fiji.

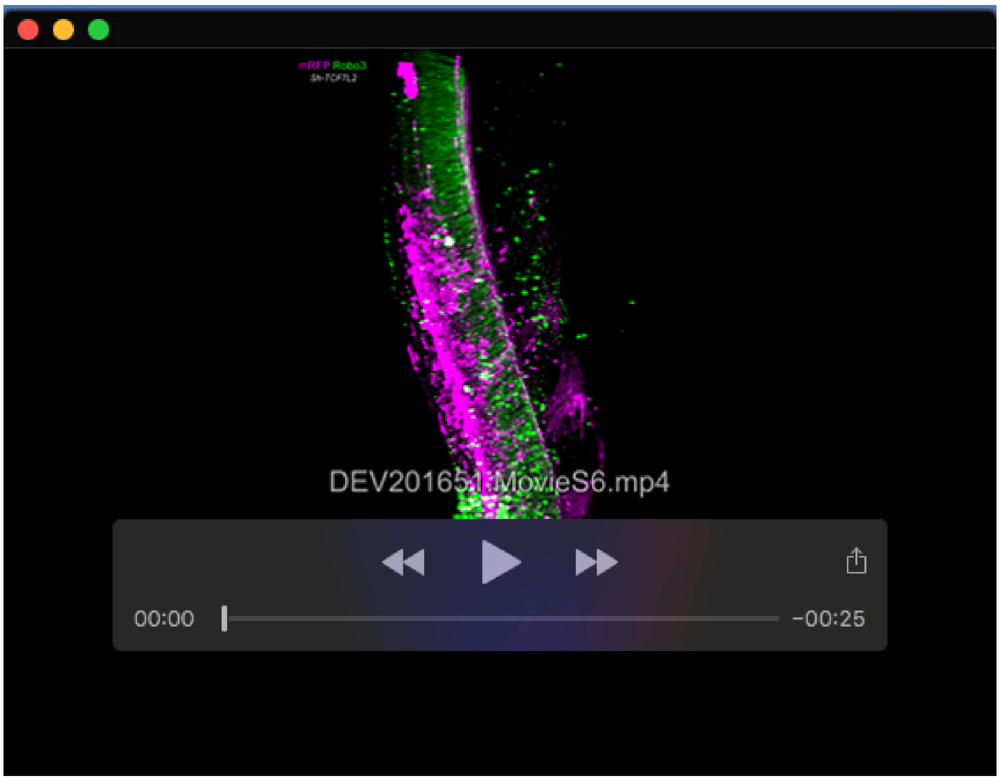

Movie 6. 360º view of 3D reconstruction of volume imaging performed through light sheet fluorescence microscopy at 9.6x of cleared E3 chick embryos electroporated for 24 h (E3 + 24 hpe) with pCS2-mRFP plus pSHIN-ShTCF7L2 that were whole-mount immuno labeled with anti RFP (magenta) and anti Robo3 (green) antibodies. The RFP channel is shown alone in grey scale after the magenta/green sequence. The image shows 1º steps of a 360º rotation with the axis placed in the cranial-caudal direction. 3D reconstruction was generated with ImageJ/Fiji.
