# Supplementary material for: Neurogenesis redirects β-catenin from adherens junctions to the nucleus to promote axonal growth
Source: Development. 2023 Aug 24;150(16):dev201651. doi: 10.1242/dev.201651 (PMC10482005; doi:10.1242/dev.201651)
Supplement: Supplementary file 1 [file develop-150-201651_review_history.pdf]

Neurogenesis redirects ß-catenin shipping address from adherens junctions to the nucleus to promote axonal growth

Antonio Herrera, Anghara Menendez, Andrea Ochoa, Lidia Bardia, Julien Colombelli and Sebastian Pons
DOI: 10.1242/dev.201651

Editor: Steve Wilson

Review timeline

Original submission:	27 January 2023
Editorial decision:	6 March 2023
First revision received:	27 June 2023
Editorial decision:	18 July 2023
Second revision received:	19 July 2023
Accepted:	26 July 2023

Original submission

First decision letter

MS ID#: DEVELOP/2023/201651

MS TITLE: Neurogenesis changes ß-catenin shipping address from adherens junctions to nucleus to booster axonal growth.

AUTHORS: Sebastian Pons, Antonio Herrera, Anghara Menendez, Andrea Ochoa, Lidia Bardia, and Julien Colombelli

I have now received all the referees' reports on the above manuscript, and have reached a decision. The referees' comments are appended below, or you can access them online: please go to BenchPress and click on the 'Manuscripts with Decisions' queue in the Author Area.

As you will see, the referees express considerable interest in your work, but have some significant criticisms and suggestions for improving your manuscript. If you are able to revise the manuscript along the lines suggested, I will be happy receive a revised version of the manuscript. Please also note that Development will normally permit only one round of major revision. If it would be helpful, you are welcome to contact us to discuss your revision in greater detail. Please send us a point-by-point response indicating your plans for addressing the refereeâ€™s comments, and we will look over this and provide further guidance.

Please attend to all of the reviewers' comments and ensure that you clearly highlight all changes made in the revised manuscript. Please avoid using 'Tracked changes' in Word files as these are lost in PDF conversion. I should be grateful if you would also provide a point-by-point response detailing how you have dealt with the points raised by the reviewers in the 'Response to Reviewers' box. If you do not agree with any of their criticisms or suggestions please explain clearly why this is so.

© 2023. Published by The Company of Biologists under the terms of the Creative Commons Attribution License (https://creativecommons.org/licenses/by/4.0/).

Reviewer 1

Advance summary and potential significance to field

This is an interesting manuscript analysing commissural axon guidance and dependency on tcf signalling. The authors show sub-cellular distribution of beta-catenin and used RNAseq to identify down-stream targets, which comprise robo adhesion molecules. Importantly, they over-express a dominant-negative wtn receptor in post-mitotic neurons to show independence of this tcf signalling role from wnt signalling. Commissural axon guidance is a well-researched field, and the authors offer additional interesting detail.

Comments for the author

A major claim is that the tcf signalling is wnt-independent. The only tool they use is a dominant-negative receptor construct they over-express under a neuronal promoter. As expected, this has no effect on axon growth. However, a technical control is missing to validate that this negative result as specific. In other words, if the construct was insufficient to inhibit wnt signalling, they would still observe the same outcome of the experiment. This means more technical controls are needed for this construct.

Additional comments:
The authors may wish to discuss this paper: “Canonical wnt signaling is required for commissural axon guidance” PMID: 26014644

“In ovo electroporation of chick NTs permits non-aggressive genetic manipulations that can be followed for almost unlimited periods during neural development.” rephrase terms such as “non-aggressive genetic manipulations” and “almost unlimited periods” are not clearly defined and are uninformative.

The material and methods part should include more detail on bias mitigation strategies, such as occluding the experimental condition during image acquisition etc. In the Statistics sections, the author should give the reasons for using the three different multiple comparison tests.

The figure legends for each graph should state what was considered an analytical unit for statistical analysis and these should also be represented as individual dots in the graphs.

“Besides, as neurogenesis extended to more ventral regions in E5 and E6, the presence of Top+ neurons also extended ventrally (Fig 1A, B).” The bluish fluorescence does not seem to label cell bodies of neurons. The Authors should indicate the cell populations they mean by arrows and maybe show higher magnifications. As it appears, the vast majority of green cells look like ventricle-contacting progenitors?

“These effects were cell-autonomous, as total ROBO3 expression measured in the descending commissural tracts at the MC level was unaffected by either treatment (Fig 2C).” This statement seems to be based on one antibody labelling, and should be moderated.

Some concluding sentences have unclear wording, such that the sense is obscured, such as in this one: “Therefore, the activation of the ß-catenin/Tcf-dependent transcription in commissural neurons shall be intrinsically promoted by the differentiation process itself and not by extrinsic Wnt proteins.” The underlined phrase is particularly unusual.

Reviewer 2

Advance summary and potential significance to field

This study by Herrera et al shows a Wnt-independent pathway for B-catenin in regulating the growth of commissural spinal axons. The authors found that, following the early Wnt-mediated Tcf-transcription during neural development, B-catenin localization changes from the adherens junctions to the nucleus after the development of commissural neurons. This localization of B-

© 2023. Published by The Company of Biologists under the terms of the Creative Commons Attribution License (https://creativecommons.org/licenses/by/4.0/).

catenin promotes Tcf-dependent transcription in a Wnt-independent manner and regulates genes implicated in axon growth, guidance and adhesion. This is a well-executed study that includes an impressive set of genetic perturbations and techniques, highlighting the power of the chick model system for identifying mechanisms of spinal cord development. Figures are well organized, and the manuscript is clearly written. Nonetheless, this reviewer has some suggestions to improve the clarity and rigor of the data presented.

Comments for the author

MAJOR COMMENTS:
1 – Cre-mediated tools are a major and elegant source of data in this study. It is therefore important to validate these tools by addressing the efficiency and specificity of the electroporation and Cre-mediated recombination assays. E.g. Quantification of S1C and S1D. Additionally, how well does endogenous expression correlate with the labeling generated via the tubb3 Cre driver? How well does endogenous Tcf-responsive expression correlate with the expression of an endogenous Tcf-responsive gene?

2 – The combination of in vivo and ex vivo approaches in Figure 2 is quite nice. But since both Tcf activation or repression cause a reduction in Tcf-mediated transcription, it is important to provide the authors' interpretation of this result. Are the defects observed dose-dependent? For instance, does modulating the timing or amount of electroporation impact these phenotypes?

MINOR COMMENTS:
3 – Sample sizes are needed for all quantifications, especially for graphs where individual data points are not shown.
4 – In the description of Figure 3, the authors state they manually selected 12 genes related to axon outgrowth and guidance. How were these genes selected? Were these the 12 most downregulated genes or were there more?
5 – The images in Figure 4E are compelling, but not quantified. In addition, what is the sample size?
6 – More labels in Figure 5J would be helpful to indicate the windows of axon outgrowth and elongation much like the labels shown on Figure 5J. It was difficult to relate the graph in Figure 5J and images in Figure 5I to the schematic shown in Figure 5K.

<u>Reviewer 3</u>

Advance summary and potential significance to field

The results shown here suggest that before the dismantling of adherens junctions, ß-catenin activity and Tcf-dependent transcription initiate and remains high during axon elongation in a Wnt-independent manner. The demonstration that TCFL2-mediated transcription is essential for axon growth in recently differentiated spinal neurons may help to clarify previous controversial studies about the canonical Wnt pathway in axon extension and guidance.

Comments for the author

In this manuscript, Herrera and collaborators investigate the role of Tcf-dependent transcription during neurogenesis and early axon extension in the dorsal spinal cord. Then, by live imaging recordings of the chick neural tube, they visualize the dinamics of betacatenin in neural progenitors and postmitotic neurons and conclude that before the dismantling of adherens junctions, ß-catenin activity and Tcf-dependent transcription initiate in a Wnt-independent manner and remains high during axon elongation. In general, the manuscript is clearly written and it is easy to follow. However, there are several points that need to be sorted out because some of the authors claims are not fully demonstrated:

1. Fig.1. The authors should provide an explanation about why there are so few GFP cells after electroporation of Tubb3::TopmGFP. I understand that only postmitotic cells are labelled but,

© 2023. Published by The Company of Biologists under the terms of the Creative Commons Attribution License (https://creativecommons.org/licenses/by/4.0/).

taking into account that most of the electroporated progenitors become postmitotic at this stage, the number of GFP cells seems to be too low.

2. The authors claim that cells from the dorsal SC undergo Tcf-dependent transcription. What is the explanation for seeing GFP fluorescence in other parts of the SC? Is this phenomenon affecting exclusively dl1neurons or other dorsal interneurons use a similar process to extend their axons?

3. Fig2. The experiments in vitro are a nice system to demonstrate that Tcf-dependent transcription is essential for axon growth. However, I do not understand why they don´t use a similar system to test the role of Betacat in postmitotic neurons. They could electroporate a conditional (flox-dependent) short hairping beta-catenin construct using the Tubb3 driver in isolated neurons and test whether the downregulation of Betacat it is really important in postmitotic neuorns to extend the axon.

4. Fig3. The information regarding Affymetrix data should be uploaded in a public database (GEO for instance). In addition, a supplementary table containing a list with the 280 and 550 DEG should be provided.

5.Also related to the analysis of the Affymetrix data, what is the criteria used to highlight what they call “manually selected genes”? It is Robo3 one of the DEG?

6. Fig. 4. Downregulation of TCF7 or TCF7L1 does not have effect on axon growth. but TCF7L2 does and therefore, this transcription factor seems to be the responsible for the activation of the TCF-dependent transcription in dorsal SC neurons. Thus, from the moment that they describe these experiments, they should refer to the TCF-dependent transcription as TCF7L2-dependent transcription.

7. In the light-sheet experiments they observe more ipsilateral axons but it is TCF7L1-transcription affecting axon growth or it is important for determining axon guidance? This is not clear. To address this question, the authors should find a way to quantify whether the total number of axons decreases or it is the ipsi/contra ratio what it changes after downregulation of TCF7L1.

8. Fig5. There is no reference to how was the LRPdn form tested. If this form is used for the first time in this paper, the authors should demonstrate that it is really working as a dominant negative form. Specially since the result obtained after its electroporation is negative. Also, it would be possible that Wnts are acting through a different combination of receptors. Since different Frizzle receptors are downregulated when the TCF-transcription is blocked, it is likely that these neurons respond to Wnt. Therefore, they should demonstrate by other means that Wnt proteins are not involved in this process.

9. Fig6F. How many cells are quantified in this experiment? In panel Fig6E’ it is difficult to visualize the translocation of betacat to the nucleus during abscission. Triple staining with DAPI or a nuclear marker should be provided.

Minor:
- There is no description of the Affymetrix microarray experiments in the Methods section.
- Previous articles reporting the role of betacatenin or other components of the Wnt signaling pathway should be incorporated into the discussion section (e.g. Aviles et al., 2016).
- There are some typos along the manuscript that should be corrected. For instance, in the abstract, line 7 it says beta-catenina.
- Pg5. Line 11, what is TK?
- Pg5. In the text, it says TRE while in the figure, the very same element is called WRE. Please unify all the terms in text and figures.
- Pg5. Please state how short is the life of the short-living GFP used in Fig1.
- It is not clearly explained how GFP FI is normalized in the experiments. A more exhaustive explanation should be provided in the methods section.

© 2023. Published by The Company of Biologists under the terms of the Creative Commons Attribution License (https://creativecommons.org/licenses/by/4.0/).

First revision

Author response to reviewers' comments

Reviewer 1 Advance Summary and Potential Significance to Field:
R
This is an interesting manuscript analysing commissural axon guidance and dependency on tcf signalling. The authors show sub-cellular distribution of beta-catenin and used RNAseq to identify down-stream targets, which comprise robo adhesion molecules. Importantly, they over-express a dominant-negative wtn receptor in post-mitotic neurons to show independence of this tcf signalling role from wnt signalling. Commissural axon guidance is a well-researched field, and the authors offer additional interesting detail.

Reviewer 1 Comments for the Author:
R
A major claim is that the tcf signalling is wnt-independent. The only tool they use is a dominant-negative receptor construct they over-express under a neuronal promoter. As expected, this has no effect on axon growth. However, a technical control is missing to validate that this negative result as specific. In other words, if the construct was insufficient to inhibit wnt signalling, they would still observe the same outcome of the experiment. This means more technical controls are needed for this construct.

A
We acknowledge the reviewer's comment regarding the need for a proper control in this experiment. To address this concern and ensure clarity, we conducted a specific experiment in which the inhibitory activity of LRP-5-6 was indisputable. For this purpose, we transfected E2 chick neural tubes for a duration of 40 hours with two constructs: TOP-H2B·RFP, which expresses nuclear RFP under a Wnt response element, and either pCIG-LRPdn, which expresses the dominant negative form of LRP5-6 along with nuclear GFP as a bicistronic element, or pCIG as a control. Our observations revealed that the expression of pCIG-LRPdn clearly inhibited the expression of TOP-H2B·RFP in the dorsal Wnt response domain and the dorsal root ganglia (DRG). These two regions are known to exhibit activation of Wnt signaling by endogenous Wnt factors in E2-E4 chick neural tubes. We have included this result in the relevant section of the results and it is also depicted in Figure S3.

Additional comments:
R
The authors may wish to discuss this paper: “Canonical wnt signaling is required for commissural axon guidance” PMID: 26014644

A
Thank you for your update. We have incorporated the paper "Aviles and Stoeckli, 2016" into the bibliography. Furthermore, in the discussion section, we have contrasted our results with their conclusions regarding the role of Lrp 5-6 in post-crossing axon guidance. This comparison is addressed in the last seven lines of the first subsection of the discussion. We appreciate your suggestion and have made the necessary amendments to address it.

R
“In ovo electroporation of chick NTs permits non-aggressive genetic manipulations that can be followed for almost unlimited periods during neural development.” rephrase terms such as “non-aggressive genetic manipulations” and “almost unlimited periods” are not clearly defined and are uninformative.

A
Done, thank you.

R
The material and methods part should include more detail on bias mitigation strategies, such as occluding the experimental condition during image acquisition etc.

© 2023. Published by The Company of Biologists under the terms of the Creative Commons Attribution License (https://creativecommons.org/licenses/by/4.0/).

A
The way we take the images when they are for quantification is by giving them a code that is only revealed after the analysis. We have now included this in methods.

R
In the Statistics sections, the author should give the reasons for using the three different multiple comparison tests.

A
In the statistical analysis, we utilized different statistical tests based on the nature of the data being compared, following the recommendations provided by the GraphPad Prism built-in assistance. These choices were made in consultation with our statistical experts, who approved the selected tests. We have now included this information in the Methods section to clarify our approach to statistical analysis. Thank you for bringing this to our attention, and we appreciate your input.

R
The figure legends for each graph should state what was considered an analytical unit for statistical analysis and these should also be represented as individual dots in the graphs.

A
In our experiments, the analytical unit was primarily a slice. To enhance clarity, we have represented the results as bar graphs displaying the mean values of different slices along with the standard error of the mean (SEM) for each condition point. It is important to note that the quantified slices were obtained from a minimum of three transfected embryos. We have updated the figure legends to include the number of slices used for each data point as well as the number of embryos from which the slices were derived. In contrast, the time-lapse results involve measurements of fluorescence intensity taken over time from a single representative cell. We have provided thorough explanations of this methodology both in the figure legends and the methods section to ensure a clear understanding of the approach. Thank you for bringing this up, and we appreciate your feedback.

R
“Besides, as neurogenesis extended to more ventral regions in E5 and E6, the presence of Top+ neurons also extended ventrally (Fig 1A, B).” The bluish fluorescence does not seem to label cell bodies of neurons. The Authors should indicate the cell populations they mean by arrows and maybe show higher magnifications. As it appears, the vast majority of green cells look like ventricle-contacting progenitors?

A
As you may already be aware, in neural progenitor cells the initial markers of differentiation become apparent even before their final division, whether it be asymmetric or neurogenic symmetric division. In later sections of the paper, we demonstrate that both the expression of b3-tubulin and the activity of TOP (Tcf Optimal Promoter) increase in neuroblast-like cells that have initiated the differentiation process. The images presented in Figure 1A are included in the paper as the initial findings that led us to speculate about the activation of TOP activity during differentiation in regions not conventionally considered Wnt response domains, specifically in regions ventral to the classic dorsal Wnt domain. Additionally, we observed that these clusters of TOP-positive cells were more prevalent in older embryos and often contained cells exhibiting morphological characteristics typical of differentiating neurons. In accordance with the reviewer's suggestion, we have now indicated with a red arrow a group of ventricular Top+ cells out of the Wnt response domain and with asterisks dorsal MZ Top+ neurons. We have revised the wording in the results section and included an explicit mention of this observation in the figure legend.

R
“These effects were cell-autonomous, as total ROBO3 expression measured in the descending commissural tracts at the MC level was unaffected by either treatment (Fig 2C).” This statement seems to be based on one antibody labelling, and should be moderated.

© 2023. Published by The Company of Biologists under the terms of the Creative Commons Attribution License (https://creativecommons.org/licenses/by/4.0/).

A
In this set of experiments, the number of transfected cells was kept sufficiently low to avoid any significant impact on the overall population of commissural neurons. To ensure that electroporation did not alter the structure of the neural tube, we conducted staining with anti- robo3 on both electroporated and non-electroporated sides. By comparing the staining patterns, we confirmed that electroporation did not disrupt the normal architecture of the neural tube. While we did not specifically demonstrate that the observed effects were independent of any other external factors, it is highly likely that the effects are primarily cell- autonomous, affecting only the electroporated cells. To address the reviewer's suggestion, we have revised the sentence to reflect this probability. The statement now reads, "These effects may be cell-autonomous" instead of "These effects were cell-autonomous." Thank you for the feedback, and we have made the necessary adjustment to the manuscript.

R
Some concluding sentences have unclear wording, such that the sense is obscured, such as in this one: “Therefore, the activation of the ß-catenin/Tcf-dependent transcription in commissural neurons shall be intrinsically promoted by the differentiation process itself and not by extrinsic Wnt proteins.” The underlined phrase is particularly unusual.

A
Thank you, we have rewritten the sentence to “ Hence, the activation of ß-catenin/Tcf- dependent transcription in pre-crossing commissural neurons is likely to be an inherent consequence of the differentiation process rather than being influenced by external Wnt proteins”

Reviewer 2 Advance Summary and Potential Significance to Field:
This study by Herrera et al shows a Wnt-independent pathway for B-catenin in regulating the growth of commissural spinal axons. The authors found that, following the early Wnt-mediated Tcf-transcription during neural development, B-catenin localization changes from the adherens junctions to the nucleus after the development of commissural neurons. This localization of B-catenin promotes Tcf-dependent transcription in a Wnt-independent manner and regulates genes implicated in axon growth, guidance and adhesion. This is a well-executed study that includes an impressive set of genetic perturbations and techniques, highlighting the power of the chick model system for identifying mechanisms of spinal cord development. Figures are well organized, and the manuscript is clearly written. Nonetheless, this reviewer has some suggestions to improve the clarity and rigor of the data presented.

Reviewer 2 Comments for the Author:
MAJOR COMMENTS:
R
1 – Cre-mediated tools are a major and elegant source of data in this study. It is therefore important to validate these tools by addressing the efficiency and specificity of the electroporation and Cre-mediated recombination assays. E.g. Quantification of S1C and S1D.

A
We employed the b3-tubulin enhancer, as described in Bergsland et al., 2011, to regulate CRE expression specifically in differentiating neurons. The specificity of this enhancer has been well-established by various research groups. In contrast, the modifications made to the pCIG vector to enable its transcriptional control by CRE expression were developed within our own laboratory and were used for the first time in this study. Initially, we visually assessed the expression of GFP from the floxed pCIG vectors and observed minimal fluorescence in the absence of CRE, which significantly increased upon b3-tubulin:CRE expression, predominantly in cells displaying neuronal morphology. However, in response to the reviewer's suggestions, we have now quantified the fluorescence intensity of GFP in neural tubes transfected with the floxed pCIG vector alone or in combination with b3-tubulin:CRE in the Tuj1+ areas (Fig S1C). Additionally, following the reviewer’s recommendation, we have performed quantification of the images presented in Fig S1D and although a similar quantification was already presented in Fig 1M, we took the opportunity to compare now the levels of Tubb3:TopTubb3::Top:GFP and Tubb3::CAG:GFP with

© 2023. Published by The Company of Biologists under the terms of the Creative Commons Attribution License (https://creativecommons.org/licenses/by/4.0/).

Top:GFP. These analyses confirmed that, unlike the dorsal-biased TOP activity observed when this tool is expressed without restrictions, TOP activity is uniformly distributed among the dorsal, medial, and ventral regions of the ventricular zone when it is specifically expressed in neurons. It is important to note that, although it is impossible to establish the precise percentage of CRE recombination using this experimental configuration due to variations in transfection efficiency, we explicitly state in the paper that none of our conclusions rely on recombination efficiency but rather on the specificity of the approach. The efficiency of recombination depends not only on the expression of CRE but also on the percentage of cells successfully transfected. We appreciate the reviewer's guidance, and we have clarified these points in the manuscript.

R
Additionally, how well does endogenous expression correlate with the labeling generated via the tubb3 Cre driver?

A
If we assume that TOP activity initiates during neural differentiation, it is indeed highly probable that TOP activity will be detected earlier with the unrestricted approach compared to the tubb3-Cre driver. This is because a certain threshold level of Cre expression is necessary before recombination can occur. Nevertheless, we consider the tubb3-Cre driver approach to be a valuable method for studying TOP activity specifically in neurons. By using the tubb3-Cre driver, we can focus on the direct effects of TOP activity in neurons during their differentiation process, without the interference of Wnt signaling present in the neural progenitor cells. This allows for a more precise understanding of the role of TOP activity in neuronal development and function. We have further clarified these points in the manuscript to highlight the advantages of the tubb3-Cre driver approach. Thank you for your suggestion.

R
How well does endogenous Tcf- responsive expression correlate with the expression of an endogenous Tcf-responsive gene?

A
The reviewer's question is indeed intriguing, and we appreciate their interest in exploring the relationship between TOP activity and the expression of classical Wnt response genes such as Lgr5 or the genes identified in our study, such as Robo1. Unfortunately, we encountered technical limitations that prevented us from obtaining satisfactory answers to this question. To address this issue, we attempted to combine fluorescent TOP-RFP imaging with in situ hybridization analysis of the aforementioned genes. However, the combination of these two techniques did not provide the necessary cellular resolution required for a proper interpretation of the results.

R
2 – The combination of in vivo and ex vivo approaches in Figure 2 is quite nice. But since both Tcf activation or repression cause a reduction in Tcf-mediated transcription, it is important to provide the authors' interpretation of this result. Are the defects observed dose-dependent? For instance, does modulating the timing or amount of electroporation impact these phenotypes?

A
I am afraid that the reviewer has misinterpreted the results presented in Fig 2. As we demonstrated in the paper by Herrera et al 2014 , the two tools used in this figure have an opposite effect on TCF-dependent transcription, TcfEnR represses it and Sb-Catenin activates it. What the figure shows is the effect of these tools on the commissural axons, and in this case we come to the conclusion that both a lack and a hyperactivation of TCF activity lead to the reduction of said axons.

Reviewer 2
MINOR COMMENTS:

R
3 – Sample sizes are needed for all quantifications, especially for graphs where individual data points are not shown.

© 2023. Published by The Company of Biologists under the terms of the Creative Commons Attribution License (https://creativecommons.org/licenses/by/4.0/).

A
In most experiments the analytical unit is an slice, for clarity we represented the results as bar-graphs showing the mean of the different slices ± standard error of the mean (SEM), calculated for each condition point; the quantified slices always came from at least 3 transfected embryos. Now we have added in the legends the number of slices used in each data point and the number of embryos used to make the slices.

R
4 – In the description of Figure 3, the authors state they manually selected 12 genes related to axon outgrowth and guidance. How were these genes selected? Were these the 12 most downregulated genes, or were there more?

A
As shown in the volcano plot of fig 2B, (the blue dots) 567 genes were significantly downregulated in this study (log2 fold change ≤ 2 with adjusted p≤0.05). As we wanted to validate the DNA Gene-Chip results by RT q-PCR and also assess whether the activation of the TCF pathway would have an opposite effect and we did not want to study the whole 567 downregulated genes, we looked for downregulated genes that appeared in the 10 top panther pathway categories and also appeared in our bibliographic review of axon guidance. Finally we decided to limit the study to 12 genes that are the ones highlighted in the Fig 2B'. From those we failed to obtain reliable RT q-PCR amplification in three of them, ALCAM, NEO and FDZ7. Thus, Fig 3C shows the results obtained with the remaining 9.

R
5 – The images in Figure 4E are compelling, but not quantified. In addition, what is the sample size?

A
These images were not originally intended for quantitative analysis due to the potential variations in fluorescence intensity measurements resulting from the 3D reconstruction process. Instead, we included them in the paper to visually illustrate the astonishing phenotype caused by TCF7L2 knockdown, as mentioned by the reviewer. The 360º images, which were not able to be loaded on the journal server due to size limitations during the initial review, will be provided as supplemental material if the manuscript is accepted for publication. Regarding the whole embryo light sheet imaging process, it is a laborious and time-consuming procedure, especially when incorporating antibody labeling. Although we labeled more embryos, we ultimately recorded data from only two embryos per treatment group, as the results were highly consistent. However, to address the reviewer's suggestions, we have utilized the original image stacks from the light sheet acquisition to generate Z projections of the planes containing the axon tracts. This allows us to compare the fluorescence intensity between the ipsilateral and contralateral tracts, and the results are presented in Figure 4F. Additionally, the sample size has been added to the figure legend to provide transparency regarding the number of embryos analysed.

R
6 – More labels in Figure 5J would be helpful to indicate the windows of axon outgrowth and elongation much like the labels shown on Figure 5J. It was difficult to relate the graph in Figure 5J and images in Figure 5I to the schematic shown in Figure 5K.

A
We assumed that the reviewer meant more labels in Fig 5I and not in 5J. Thus, following reviewer's suggestion we have now added new labels indicating the apical abscission and axon outgrowth times to the images of Fig 5I.

Reviewer 3 Advance Summary and Potential Significance to Field:

The results shown here suggest that before the dismantling of adherens junctions, ß-catenin activity and Tcf-dependent transcription initiate and remains high during axon elongation in a

© 2023. Published by The Company of Biologists under the terms of the Creative Commons Attribution License (https://creativecommons.org/licenses/by/4.0/).

Wnt-independent manner. The demonstration that TCFL2-mediated transcription is essential for axon growth in recently differentiated spinal neurons may help to clarify previous controversial studies about the canonical Wnt pathway in axon extension and guidance.

Reviewer 3 Comments for the Author:
In this manuscript, Herrera and collaborators investigate the role of Tcf-dependent transcription during neurogenesis and early axon extension in the dorsal spinal cord. Then, by live imaging recordings of the chick neural tube, they visualize the dinamics of betacatenin in neural progenitors and postmitotic neurons and conclude that before the dismantling of adherens junctions, ß-catenin activity and Tcf-dependent transcription initiate in a Wnt- independent manner and remains high during axon elongation. In general, the manuscript is clearly written and it is easy to follow. However, there are several points that need to be sorted out because some of the authors claims are not fully demonstrated:

R
1. Fig.1. The authors should provide an explanation about why there are so few GFP cells after electroporation of Tubb3::TopmGFP. I understand that only postmitotic cells are labelled but, taking into account that most of the electroporated progenitors become postmitotic at this stage, the number of GFP cells seems to be too low.

A
The Top:LoxP-mGFP vector is a new version of the widely used Top:Flash vector (upstate # 21-170) and its fluorescent variants GFP and RFP (Herrera et al, 2014). This tool has proven to be reliable for assessing TCF-dependent transcriptional activity in different cell systems and cell types, including neural progenitors. The version presented in this study consists of 5 repeats of TCF binding sites, a minimal promoter, a floxed polyA sequence, and the coding sequence of mGFP. When combined with the Tubb3:Cre vector, the polyA sequence is excised in postmitotic cells during neuronal differentiation. Consequently, after recombination, only postmitotic cells with activated TCF-dependent transcription can express mGFP.

As pointed out by the reviewer, and as supported by our control (Tubb3::CAG:mGFP), the majority of progenitors become postmitotic at this stage, but only a subset of them activate TCF-dependent transcription (Tubb3::Top:mGFP+ cells). Our results indicate that these cells are contralateral interneurons in the early stage of differentiation. This is demonstrated by the fact that nearly 100% of the axons in the SC commissure express Tubb3::Top:mGFP, and this percentage remains residual in the axons of the contralateral VF and LF (Fig 1N).

R
2. The authors claim that cells from the dorsal SC undergo Tcf-dependent transcription. What is the explanation for seeing GFP fluorescence in other parts of the SC? Is this phenomenon affecting exclusively dl1neurons or other dorsal interneurons use a similar process to extend their axons?

A
TCF-dependent transcription is known to be activated by neural progenitors in the dorsal SC (Fig 1A, Alvarez-Medina et al, 2008), however, as the reviewer rightly indicates, we have observed that during neuronal differentiation, TCF-dependent transcription is also activated in different postmitotic populations along the D-V axis (Fig 1I). Again, the fact that nearly 100% of the axons in the SC commissure are GFP+ indicates that TCF-dependent transcription is not exclusively activated by dl1 neurons during differentiation. The activation observed in the more medial spinal cord could be attributed to the differentiation of contralateral interneurons such as dI5 and dI6, while the ventral signal may be explained by the differentiation of ventral contralateral interneurons like V0 and V3. Therefore, our findings suggest that TCF-dependent transcription plays a role in the differentiation of various postmitotic populations along the D-V axis, including both dorsal and ventral contralateral interneurons.

R
3. Fig2. The experiments in vitro are a nice system to demonstrate that Tcf-dependent transcription is essential for axon growth. However, I do not understand why they don´t use a similar system to test the role of Betacat in postmitotic neurons. They could electroporate a

© 2023. Published by The Company of Biologists under the terms of the Creative Commons Attribution License (https://creativecommons.org/licenses/by/4.0/).

conditional (flox-dependent) short hairping beta-catenin construct using the Tubb3 driver in isolated neurons and test whether the downregulation of Betacat it is really important in postmitotic neuorns to extend the axon.

A
We conducted the experiment presented in Figure 2E and D to investigate the necessity of Tcf-dependent transcription for axon elongation in an isolated environment. This experiment served as a complement to the findings depicted in Figure 2A, where we observed that TcfEnR expression in the neural tube context appeared to reduce axon length in transfected cells. The objective of this experiment was to separate the effects of guidance and elongation. The results clearly demonstrated that axon elongation was affected regardless of guidance cues. Although a similar experiment could have been performed using CRE-LOX-controlled ShRNA expression to examine the requirement of ß-catenin for axon elongation in vitro, we decided against it for several reasons. Firstly, CRE-LOX-controlled ShRNA expression is not as commonly used as CRE-LOX-controlled protein expression, possibly due to the limited effectiveness of transcription insulator cassettes for RNA polymerase III, which is utilized by promoters producing ShRNAs (such as the U6 promoter). Additionally, we aimed to inhibit Tcf-dependent transcription as early as possible during differentiation, and a ShRNA approach would have delayed the effect until the accumulated ß-catenin was depleted. Finally, in our recent publication (Herrera et al., 2021), we employed ShRNAs targeting ß-catenin and observed alterations in cell polarity upon its depletion, therefore the use of his strategy would make it difficult to interpret the contribution of b-catenin transcriptional role to the phenotype.

R
4. Fig3. The information regarding Affymetrix data should be uploaded in a public database (GEO for instance). In addition, a supplementary table containing a list with the 280 and 550 DEG should be provided.

A
The Affymettrix data are now in GEO, accession number GSE234518 (Expression data of Tubb3+ cells from E4 chicken neural tube). The dataset will be available from Jun 27, 2023 (https://www.ncbi.nlm.nih.gov/geo/query/acc.cgi?acc=GSE234518). To review GEO accession GSE234518 before the release date, please go to
https://www.ncbi.nlm.nih.gov/geo/query/acc.cgi?acc=GSE234518, entering the token qtyramccttoxler into the box.
Following the reviewer's recommendation, we have included two supplementary tables, one that provides a comprehensive list of genes that exhibit upregulation and downregulation in response to the suppression of Tcf activity and a second one containing the metadata of the DNA-array analysis. In addition, we would like to apologize for an error in the previous version of the manuscript. Upon reviewing our data, we realized that the actual total number of upregulated and downregulated genes differs from what was previously stated. The corrected figures indicate that we observed 302 upregulated genes and 567 downregulated genes in response to the suppression of Tcf activity. We have rectified this discrepancy in Figure 3B and also updated the corresponding information in the results section of the manuscript.

R
5.Also related to the analysis of the Affymetrix data, what is the criteria used to highlight what they call "manually selected genes"? It is Robo3 one of the DEG?

A
As indicated in the volcano plot of Figure 2B (represented by blue dots), a total of 567 genes were found to be significantly downregulated in this study (with a log2 fold change ≤ 2 and adjusted p ≤ 0.05). To validate the results obtained from the DNA Gene-Chip, as well as to investigate the potential opposite effects of TCF pathway activation, we focused on downregulated genes that belonged to the top 10 Panther pathway categories and were also identified in our literature review on axon guidance. Ultimately, we decided to narrow down our study to 12 highlighted genes shown in Figure 2B, excluding ALCAM, NEO, and FZD7 due to unreliable RT-qPCR amplification. The remaining 9 genes are presented in Figure 3C. Notably, Robo3 was not among the downregulated genes.

© 2023. Published by The Company of Biologists under the terms of the Creative Commons Attribution License (https://creativecommons.org/licenses/by/4.0/).

R
6. Fig. 4. Downregulation of TCF7 or TCF7L1 does not have effect on axon growth. but TCF7L2 does and therefore, this transcription factor seems to be the responsible for the activation of the TCF-dependent transcription in dorsal SC neurons. Thus, from the moment that they describe these experiments, they should refer to the TCF-dependent transcription as TCF7L2- dependent transcription.

A
We appreciate the reviewer's suggestion, but we believe using different terms along the manuscript would create confusion, specially in the discussion section . Therefore, we have decided to maintain consistency and clarity by sticking with the original term.

R
7. In the light-sheet experiments they observe more ipsilateral axons but it is TCF7L1-transcription affecting axon growth or it is important for determining axon guidance? This is not clear. To address this question, the authors should find a way to quantify whether the total number of axons decreases or it is the ipsi/contra ratio what it changes after downregulation of TCF7L1.

A
We have taken note that the reviewer intended to refer to TCF7L2 rather than TCFL1. Consequently, in response to the reviewer's suggestion, we measured the mRFP fluorescence intensity specifically in the axons of the ipsilateral and contralateral ventral funiculus. This measurement was conducted in both control embryos and embryos transfected with ShTCFL2. For each condition, we analyzed two embryos and calculated the ratio of fluorescence intensities between the ipsilateral and contralateral ventral funiculus. The resulting data is now presented in the form of a bar graph in Figure 4F.

R
8. Fig5. There is no reference to how was the LRPdn form tested. If this form is used for the first time in this paper, the authors should demonstrate that it is really working as a dominant negative form. Specially since the result obtained after its electroporation is negative. Also, it would be possible that Wnts are acting through a different combination of receptors. Since different Frizzle receptors are downregulated when the TCF-transcription is blocked, it is likely that these neurons respond to Wnt. Therefore, they should demonstrate by other means that Wnt proteins are not involved in this process.

A
We acknowledge the reviewer's comment regarding the need for a proper control in this experiment. To address this concern and ensure clarity, we conducted a specific experiment in which the inhibitory activity of LRP-5-6 was indisputable. For this purpose, we transfected E2 chick neural tubes for a duration of 40 hours with two constructs: TOP-H2B·RFP, which expresses nuclear RFP under a Wnt response element, and either pCIG-LRPdn, which expresses the dominant negative form of LRP5-6 along with nuclear GFP as a bicistronic element, or pCIG as a control. Our observations revealed that the expression of pCIG-LRPdn clearly inhibited the expression of TOP-H2B·RFP in the dorsal Wnt response domain and the dorsal root ganglia (DRG). These two regions are known to exhibit activation of Wnt signaling by endogenous Wnt factors in E2-E4 chick neural tubes. As the reviewer states, other Wnts can act on Frizzled receptors, but it has not been described that TCF transcriptional activity can be activated by another Wnt pathway other than the canonical Wnt pathway, where LRP5/6 is an essential co- receptor for activation. Therefore, our results with dnLRP rule out the hypothesis that TCF- dependent transcription can be activated through the Wnt/Frizzled pathway. We have included this result in the relevant section of the results and it is also depicted in Figure S3.

R
9. Fig6F. How many cells are quantified in this experiment? In panel Fig6E' it is difficult to visualize the translocation of betacat to the nucleus during abscission. Triple staining with DAPI or a nuclear marker should be provided.

© 2023. Published by The Company of Biologists under the terms of the Creative Commons Attribution License (https://creativecommons.org/licenses/by/4.0/).

A
The line graph shown in Figure 6F represents the quantification of a single cell, specifically the one depicted in Figure 6E (Movie 3). A similar experimental setup is used in Figure S3 (Movie 4). These experiments were conducted ex-vivo and involved time-lapse recordings of RFP and GFP channels. Since DAPI staining is not feasible for in vivo experiments, we employed the nuclear-targeted GFP expressed by pCIG to visualize the nucleus. Alternatively, we could have utilized a far-red nuclear-targeted protein; however, this would have required increased irradiation time. Therefore, we opted to use only RFP and GFP to optimize tissue preservation. Moreover, a clear correlation of the nuclear translocation of b-catenin in TopRFP+ cells is already shown in Fig 5F.

Minor:
R
- There is no description of the Affymetrix microarray experiments in the Methods section.

A
We apologize about that, we have now added a detailed description of the procedure followed in the Methods section under the title “Affymetrix GeneChip arrays”.

R
- Previous articles reporting the role of betacatenin or other components of the Wnt signaling pathway should be incorporated into the discussion section (e.g. Aviles et al., 2016).

A
Thank you for your update. We have incorporated the paper "Aviles and Stoeckli, 2016" into the bibliography. Furthermore, in the discussion section, we have contrasted our results with their conclusions regarding the role of Lrp 5-6 in post-crossing axon guidance. This comparison is addressed in the last seven lines of the first subsection of the discussion. We appreciate your suggestion and have made the necessary amendments to address it.

R
- There are some typos along the manuscript that should be corrected. For instance, in the abstract, line 7 it says beta-catenina.

A
Thanks it has been corrected. we have now double checked the whole manuscript to find more typos.

R
- Pg5. Line 11, what is TK?

A
Thank you, TK means Tyrosine Kinase, a definition has been added.

R
- Pg5. In the text, it says TRE while in the figure, the very same element is called WRE. Please unify all the terms in text and figures.

A
Thank you, the term TRE has been changed by WRE in the text.

R
- Pg5. Please state how short is the life of the short-living GFP used in Fig1.

A
The dGFP that we used in Fig S1B is the one described in Li et al., 1998. In that study, the authors calculated that the half-life of this form of GFP was approximately 2 hours. Aditionally, a more recent publication from Perrimon's lab (He et al., 2019) compared the half-life of dGFP with the wild-type GFP, resulting in calculated half-lives of 2 hours and 20 hours, respectively. Therefore, we believe it is prudent to state that the half-life of dGFP is around ten times shorter than that of the wild-type form. We have added the two mentioned references and the half-live of dGFP in the

© 2023. Published by The Company of Biologists under the terms of the Creative Commons Attribution License (https://creativecommons.org/licenses/by/4.0/).

corresponding paragraph in the results section.

R
- It is not clearly explained how GFP FI is normalized in the experiments. A more exhaustive explanation should be provided in the methods section.

A
We sincerely apologize for providing a confusing description of the method used to quantify and normalize GFP in the axons. The main objective of this normalization is to mitigate the effects of different transfection levels. Therefore, in each section, the fluorescence of the different compartments, namely commissure and funiculi, is normalized (after subtracting the background) by the fluorescence in the somas of the commissural neurons that project those axons. These regions are defined using either Robo3 or Tuj1 staining.

We have now rewritten the method, aiming to make it as clear as possible and explicitly stating the formula used. Once again, we apologize for any confusion caused.

Second decision letter

MS ID#: DEVELOP/2023/201651

MS TITLE: Neurogenesis changes ß-catenin shipping address from adherens junctions to nucleus to booster axonal growth.

AUTHORS: Antonio Herrera, Anghara Menendez, Andrea Ochoa, Lidia Bardia, Julien Colombelli, and Sebastian Pons

I have now received all the referees reports on the above manuscript, and have reached a decision. The referees' comments are appended below, or you can access them online: please go to BenchPress and click on the 'Manuscripts with Decisions' queue in the Author Area.

Two of the reviewers are happy with your revisions but one has one further issue for you to address relating to the graphs you present. In addition to the "Comments for the authors" explaining this, the referee indicated in comments to the editor that "all graphs in the paper need proper statistical analysis" and that you need to discuss the new graph within the text. Could you please attend to the these comments in your revised manuscript.

Reviewer 1

Advance summary and potential significance to field

as before

Comments for the author

I thank the authors for thoroughly addressing my suggestions - I have no further comments.

Reviewer 2

Advance summary and potential significance to field

This is an overall well-executed study that shows a Wnt-independent pathway for B-catenin in regulating the growth of commissural spinal axons.

© 2023. Published by The Company of Biologists under the terms of the Creative Commons Attribution License (https://creativecommons.org/licenses/by/4.0/).

Comments for the author

The revised manuscript has successfully addressed my comments and concerns.

Reviewer 3

Advance summary and potential significance to field

N/A

Comments for the author

The authors have addressed most of my concerns. However, there are still some aspects regarding the new graph they have included in Figure 4F that should be clarified: 1/ Whether the different ratio they observe between ipsi and contralateral axons in the control versus shTCF7L2 electroporated embryos is significant, and 2/ Whether that difference in the ratio is due to a lower number of contralateral axons or an increase in ipsilateral axons. This is important because if the result reflects the first hypothesis, it would mean that the commissural axons stop growing, while in the second case, it would imply that they are changing their laterality, thus affecting not only axonal growth but also axonal guidance. In either case, the should discuss the result in the context of the rest of the paper.

Second revision

Author response to reviewers' comments

Dear Steve, we have now included the requested statistics for Fig 4F and added a new paragraph to clarify that the disappearance of contralateral tracks upon TCF7L2 knockdown is a result of impaired commissural axon growth, rather than a redirection to the ipsilateral tract. Additionally, we have thoroughly reviewed all figures to ensure they have the appropriate statistics. Below, I have reproduced your initial request and the reviewers' comments, along with our responses.

Best wishes, Sebastian.

Your coment:

Two of the reviewers are happy with your revisions but one has one further issue for you to address relating to the graphs you present. In addition to the "comments for the authors" explaining this, the referee indicated in comments to the editor that "all graphs in the paper need proper statistical analysis" and that you need to discuss the new graph within the text. Could you please attend to the these comments in your revised manuscript.

R

Reviewer 3 Comments for the Author:
The authors have addressed most of my concerns. However, there are still some aspects regarding the new graph they have included in Figure 4F that should be clarified: 1/ Whether the different ratio they observe between ipsi and contralateral axons in the control versus shTCF7L2 electroporated embryos is significant, and 2/ Whether that difference in the ratio is due to a lower number of contralateral axons or an increase in ipsilateral axons. This is important because if the result reflects the first hypothesis, it would mean that the commissural axons stop growing, while in the second case, it would imply that they are changing their laterality, thus affecting not only axonal growth but also axonal guidance. In either case, the should discuss the result in the

© 2023. Published by The Company of Biologists under the terms of the Creative Commons Attribution License (https://creativecommons.org/licenses/by/4.0/).

context of the rest of the paper.

A

Following the reviewers' suggestions, we have added statistics to Fig 4F. Additionally, we have provided a more detailed explanation of the results presented in this graph within the main text. We made efforts to clarify that the decrease in contralateral axons observed in TCF7L2 knockdown is primarily due to a deficiency in the growth of commissural axons rather than a redirection towards ipsilateral tracts. Our assessment is based on the observation that in TCF7L2 knockdown embryos, as observed in the other similar experiments presented in the work, there is a significant accumulation of GFP in the somas of transfected cells, whereas no such accumulation is observed in the ipsilateral tracts. It is worth noting that the appearance of the ipsilateral tracts in TCF7L2 knockdown embryos is strikingly similar to the control ones. However, direct comparison between the two is not feasible as they belong to two different transfections. Furthermore, while the reviewer's hypothesis that TCF7L2 may not only enhance axon growth but also play a role in axon guidance is intriguing, we acknowledge that this aspect falls beyond the scope of the current study. Thus, we believe it warrants further investigation in future research endeavours.

Third decision letter

MS ID#: DEVELOP/2023/201651

MS TITLE: Neurogenesis changes β-catenin shipping address from adherens junctions to nucleus to booster axonal growth.

AUTHORS: Antonio Herrera, Anghara Menendez, Andrea Ochoa, Lidia Bardia, Julien Colombelli, and Sebastian Pons
ARTICLE TYPE: Research Article

I am happy to tell you that your manuscript has been accepted for publication in Development, pending our standard ethics checks.

© 2023. Published by The Company of Biologists under the terms of the Creative Commons Attribution License (https://creativecommons.org/licenses/by/4.0/).
